# Study on tensile mechanical response and microstructure of polypropylene fiber reinforced loess under freezing

**DOI:** 10.1371/journal.pone.0350932

**Published:** 2026-06-09

**Authors:** Yuxing Wang, Chunhui Liu, Yimin Zhong, Erqing Mao, Fei He

**Affiliations:** 1 The Highway Development Center Of Gansu Province, Lanzhou, China; 2 School of Civil Engineering, Lanzhou Jiaotong University, Lanzhou, China; Jazan University College of Engineering, SAUDI ARABIA

## Abstract

Tensile strength is one of the key parameters in the mechanics of frozen ground, widely applied in the engineering design of frozen ground and theoretical research on frost heave. Fiber-reinforced soil technology has attracted considerable attention from numerous researchers. This study utilised a custom-designed tensile testing apparatus to investigate the influence of polypropylene fiber content, freezing temperature, and loading rate on the mechanical response of frozen loess. Combined with microscopic structural observations, the research elucidates the fiber reinforcement mechanism. Experiments revealed that fiber incorporation transformed the soil’s failure mode from brittle fracture to ductile failure, characterised by multiple fissures, significantly enhancing the material’s load-bearing capacity and deformation properties. Optimal reinforcement was achieved at a fiber content of 0.3%, with tensile strength increasing by 71.4% compared to unfibered soil. The stress-strain response exhibited sustained strengthening characteristics. Tensile strength exhibits exponential growth with decreasing freezing temperatures and linear increase with rising loading rates. The incorporation of fibers further enhances the material’s response capability under dynamic loading conditions. Microstructural analysis indicates that an appropriate fiber content effectively fills soil pores and forms a spatial network structure, whereas excessive fibers cause agglomeration due to uneven distribution, resulting in structural weakening. This research provides experimental evidence and theoretical reference for the design and application of fiber-modified frozen ground in engineering projects within cold regions

## 1. Introduction

The loose granular structure of unfrozen loess exhibits significantly lower tensile strength than its compressive and shear strengths, leading to its frequent neglect in conventional engineering practice. However, the cementing effect of ice crystals on soil particles during the freezing process markedly enhances its tensile properties, rendering it an indispensable consideration in permafrost engineering design [1–3]. It is noteworthy that the tensile strength of frozen soil is closely related to engineering issues such as subgrade instability, pavement cracking, and tensile cracks at the top of slopes [4,5]. Therefore, deepening research into the tensile strength of frozen soil not only holds significant theoretical value but also carries urgent practical significance for engineering disaster prevention and mitigation.

Testing methods for the tensile strength of frozen soil are primarily categorised into direct and indirect approaches [6–8]. Direct methods encompass uniaxial and triaxial tensile testing. Within uniaxial tensile testing, universal testing machines were among the earliest instruments employed for researching the tensile strength of remoulded silt [9]. However, this method demands high precision in specimen preparation and reliable fixture connections. Particularly under frozen conditions, specimens are prone to stress concentration at the gripping ends or local defects, thereby compromising the accuracy and stability of test results [10]. Consequently, researchers have improved the testing methodology through the independent design of moulds and gripping devices [11–14]. The triaxial tensile method has also been employed to test the tensile strength of frozen soft clays [15,16]. However, the complexity of triaxial testing apparatus and principles often precludes obtaining ideal single tensile failure modes during trials, with outcomes typically manifesting as pure tensile failure, failure after shear elongation, or pure shear failure. These failure modes compromise the stability of test results. Furthermore, due to equipment limitations, key mechanical responses during failure are difficult to observe effectively [17]. Indirect methods primarily include the splitting method and the soil beam bending method [18,19]. The splitting method is suitable for brittle materials but has limited applicability for soil tensile strength, as its assumed stress state deviates from the actual tensile behaviour of frozen soil [20]. The soil beam bending method, meanwhile, involves large specimen dimensions, making uniformity difficult to guarantee, and is prone to stress displacement during testing [21]. Consequently, existing research on testing methods and apparatus for determining the tensile strength of frozen ground still exhibits shortcomings.

Against this backdrop, alongside experimental methodologies, research into enhancing the tensile properties of frozen ground through material modification has progressively emerged as a significant approach in cold region engineering studies. Multiple investigations indicate that incorporating additives such as cement, lime, fly ash, and fibers into the soil matrix can improve its mechanical properties and increase tensile strength to a certain extent [22–26]. Fibers have garnered particular attention due to their capacity to form spatial reinforcement networks within the soil matrix, significantly enhancing its toughness, ductility, and crack resistance. Common fiber types include natural fibers such as straw [27] and jute [28], synthetic fibers like geotextiles [29] and polypropylene fibers [30], as well as mineral fibers including glass fibers [31,32], basalt fibers [33,34], and steel fibers [35]. Compared to others, polypropylene fibers possess advantages such as high tensile strength, excellent chemical corrosion resistance, strong dispersibility, and relatively low cost, making them the most widely applied fiber material in soil reinforcement. Extensive research under ambient conditions demonstrates that polypropylene fibers effectively enhance soil shear strength and unconfined compressive strength, while inhibiting the initiation and propagation of tensile cracks through bridging effects. Relevant experimental results further indicate that fiber incorporation significantly increases peak tensile strength and modifies brittle tensile failure modes, imparting more pronounced ductile characteristics to the soil [36–39].

Although the mechanical properties of fiber-modified soils have been studied relatively systematically under ambient temperature conditions, the applicability of such research to engineering environments in cold regions remains inadequate. During low-temperature freezing, phase transitions occur within the soil matrix. The formation of ice crystals not only introduces additional cementing effects but is also accompanied by the dynamic evolution of unfrozen water film thickness and structural rearrangement induced by freeze-thaw cycles. This results in the soil’s microstructure and mechanical response exhibiting significant differences from those under ambient conditions [40–42]. Furthermore, existing experimental equipment for frozen soil tensile testing often faces challenges in securing specimens, preventing localized damage, and accommodating fiber-frozen soil composites. These limitations hinder the accurate characterization of the tensile failure mechanisms and mechanical responses of fiber-reinforced soils in frozen environments. To address these issues, this study independently designed and developed a dedicated frozen soil tensile testing apparatus that ensures effective specimen clamping and minimizes stress concentration during loading. Using this apparatus, a systematic series of tensile tests was conducted on polypropylene fiber-modified loess under frozen conditions. Combined with microstructural analysis, the mechanism by which fibers enhance the tensile performance of frozen soil was elucidated. Furthermore, the study investigated the evolutionary patterns and microstructural characteristics of tensile properties in fiber-reinforced loess during freezing, aiming to provide theoretical underpinnings and technical guidance for engineering design and construction in cold regions.

## 2. Experimental design

### 2.1. Testing apparatus

**Fig 1:**
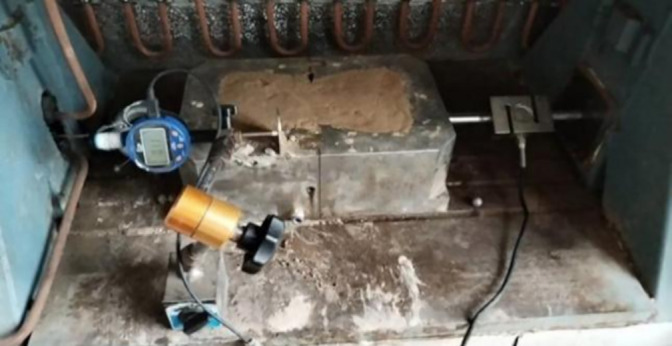
Tension box.

**Fig 2:**
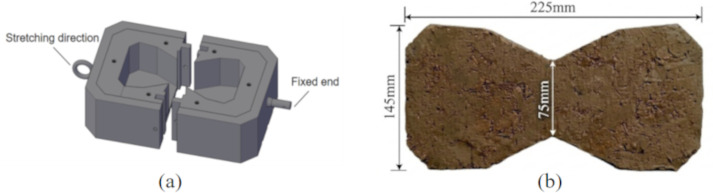
(a) Overall design of the tensile testing box and (b) schematic diagram (units: mm).

The testing apparatus utilises a tensile testing machine that has been self-developed, as illustrated in Fig 1. The tensile testing machine comprises a test bench, tensile chamber, force transmission system, cryogenic control unit, load traction mechanism, and data measurement and acquisition system. The overall dimensions of the tensile box are 300 mm × 200 mm × 105 mm. The internal chamber of the tensile box features a butterfly-shaped tensile interface formed by geometric contraction, with a total volume of 1,840 cm³. The geometric dimensions of the specimen held inside the box are 225 mm × 145mm × 75 mm, as shown in Fig 2. During the tensile test, the critical tensile longitudinal cross-section of the specimen (i.e., the butterfly-shaped contact surface) measures 75 mm × 75 mm. The force transmission system under consideration comprises a synchronous pulley with an inner diameter of 16 mm and a groove width of 25 mm, along with steel strands. Two grooves are machined into the lower section of the tensile chamber, forming a slideway in conjunction with the grooves and steel columns on the lower test bench. The load traction system is composed of a servo motor system, a reduction gearbox, and a synchronous pulley. The servo motor system comprises the following components: a servo motor, motor base, driver, data cables (pulse line, power line, and encoder line), and servo motor speed controller, achieving a minimum loading rate of 1.57 mm/min. The temperature control system consists of a liquid bath circulation chiller and a low-temperature control cabinet. The liquid bath circulation chiller employs a TMS8037-R40 medium-sized vertical precision high/low-temperature constant-temperature circulating liquid bath, with a temperature control range of −40 to +90°C and an accuracy of ±0.05°C. The internal dimensions of the low-temperature control box are 760 mm × 510 mm × 520 mm. The installation of polyurethane foam boards and heat insulation cotton is conducted on the inner and outer sides, with refrigeration copper pipes connected with the liquid bath circulation refrigeration device distributed around the inner side. The tensile force measurement system is composed of an S-type load cell and bespoke force monitoring software. The load cell has a maximum measurement limit of 1000 kg, with a precision of 0.1 kg and a data acquisition frequency of 1 s. The measurement of temperature is achieved through the utilisation of PT100 thermistors, which offer an accuracy of 0.1°C. The data is collected by AT4708V series multi-channel temperature testers, with measurements taken at 10-minute intervals. Displacement data is obtained via displacement sensors, a hub, and a displacement acquisition system. The displacement sensor utilises an Eee dial gauge, which possesses a measurement range of 0–50 mm, an accuracy of 0.01 mm, and a sampling frequency of 1 s.

### 2.2. Test soil and specimen preparation

**Fig 3:**
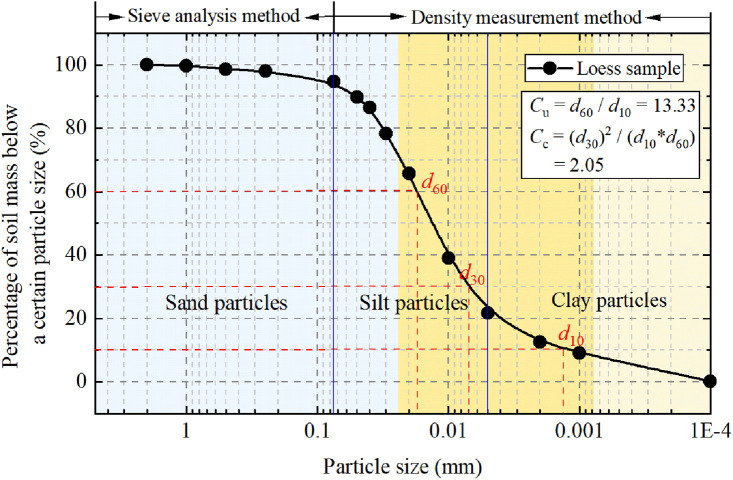
Soil particle gradation curve.

The soil samples used in this study were collected from Daqingshan in Anning District, Lanzhou, China, at depths of 0.5–2.0 m below the surface; they belong to the Quaternary Lanzhou Loess. The soil displayed a brownish-yellow hue, characterised by uniformly dispersed particles. Following the process of air-drying, the loess was subjected to sieving through a 2 mm mesh, with the intention of subsequent utilisation. The soil sample exhibited a liquid limit of 30.1%, a plastic limit of 19.5%, a plasticity index of 10.6, a specific gravity of 2.70, a maximum dry density of 1.86 g/cm³, and an optimum moisture content of 16.2%. As demonstrated in Fig 3, the gradation curve classifies the substance as a silty clay.

The polypropylene fibers used in the test were bundled filaments with a specific gravity of 0.91, a tensile strength exceeding 358 MPa, and an elastic modulus exceeding 3.5 GPa. They measured 12 mm in length with a fiber diameter of 18–48 µm and exhibited no water absorption.

Before testing, a layer of low-temperature lubricant oil was applied to the contact surfaces of the tensile testing apparatus. According to the experimental requirements, a specified amount of loess sample was weighed and placed in a container. The required fiber mass was then calculated based on the fiber content and soil mass ratio. Manually untangle the bundled fibers, then gradually mix them with dry soil using a mechanical mixer for 5 minutes. Next, add water to achieve the predetermined moisture content and mix thoroughly for 5 minutes, followed by allowing the soil to stand under sealed conditions for 12 hours. The specimen must be placed in the tensile testing box in three layers. Temperature sensors are inserted into the sample during the filling process. Scrape between layers; after backfilling, wrap the specimen’s surface with cling film. For constant-temperature freezing, Place the tensile test specimen in a low-temperature chamber pre-cooled to the target temperature and maintain it at that temperature for 24 hours. When the specimen temperature satisfies the requirements, perform the tensile test. After the tensile test had finished, a soil cutting knife was used to extract a sample from the centre. This sample was then sectioned into 5 mm × 5 mm × 20 mm specimens, which were kept frozen. SEM analysis was conducted using a ZEISS Gemini SEM 500 field emission scanning electron microscope. During testing, break off a soil sample, select a fresh fracture surface, deposit a gold coating using an ion sputtering apparatus, and capture SEM images.

The test conditions are shown in Table 1. For each test condition, three replicate tests (N = 3) were conducted, and the deviation between the individual strength values and the average strength value for the three replicates should not exceed 8% to ensure the reliability of the data. The data shown in the figure represent the average strength values from the replicate tests.

**Table 1:** Tensile strength test conditions.

Polypropylene fiber content(%)	Moisture content(%)	dry density(g/cm^3^)	Freezing temperature(℃)	Loading rate(mm/min)
0	16.2	1.80	−2、-3、-5	1.57
0	16.2	1.80	−2、-3、-5	4.71
0	16.2	1.80	−2、-3、-5	7.85
0.2	16.2	1.80	−5	1.57
0.3	16.2	1.80	−1、-2、-3、-5	1.57
0.3	16.2	1.80	−5	4.71
0.3	16.2	1.80	−5	7.85
0.4	16.2	1.80	−5	1.57
0.5	16.2	1.80	−5	1.57

### 2.3. Equipment validation

**Fig 4:**
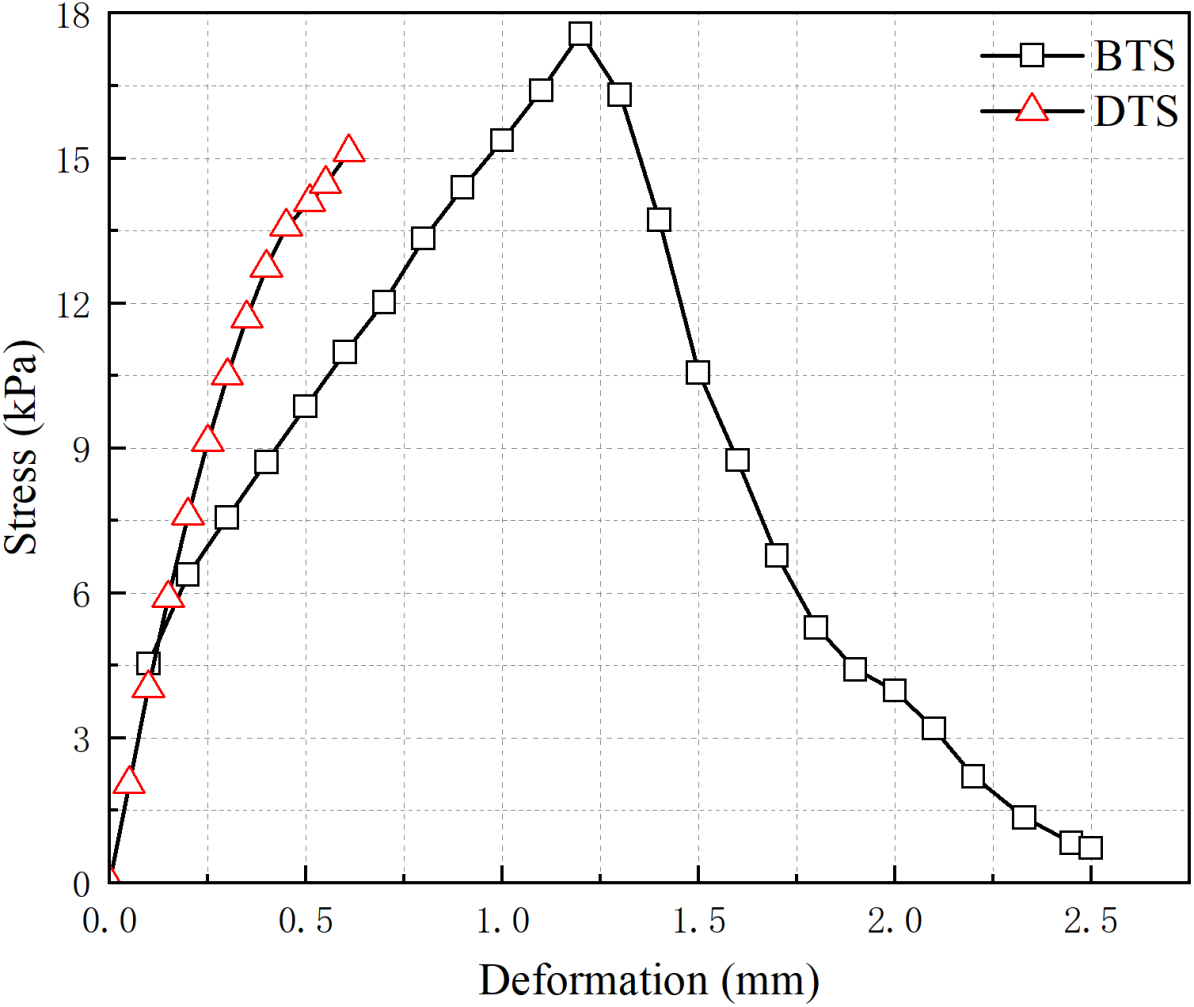
Stress-strain curves of loess under different testing methods.

To verify the accuracy of the self-developed tensile testing machine, comparative experiments were conducted in this study. The reliability of the device was evaluated by comparing the results obtained from the tensile testing machine with those from the classic Brazilian splitting test. Under room temperature conditions (20°C), three sets of parallel experiments were conducted using the two methods described above to measure the tensile strength of loess samples (moisture content 16.2%, dry density 1.8 g/cm³). The data shown in the figure represent the average strength values from the parallel experiments. As shown in Fig 4, the peak tensile strength measured by the self-developed tensile testing machine was 15.12 kPa, while the peak strength measured by the classic Brazilian splitting test method was 17.58 kPa; the former was 86% of the latter. The relationship between the data from the uniaxial tensile test in this study and the classic Brazilian splitting test is similar to the results of previous studies [43,44].This is because, in the classic Brazilian splitting test, the combined tensile-compressive stress state within the specimen and the end-face friction effect inhibit the free propagation of microcracks, whereas the self-developed tensile testing machine performs uniaxial tensile testing, reducing the influence of compressive stress; macroscopic fracture always initiates first at localized microdefects or large voids within the specimen. Therefore, due to the combined influence of composite tensile-compressive stress constraints and end-face friction boundary effects, results obtained from the classic Brazilian split test are often higher than those from uniaxial tensile testing. This confirms that the self-developed tensile testing apparatus can effectively avoid interference from boundary compression effects and is a reliable method for obtaining true uniaxial tensile strength parameters of loess.

## 3. Analysis of test results

### 3.1. Failure modes of test specimens

**Fig 5:**
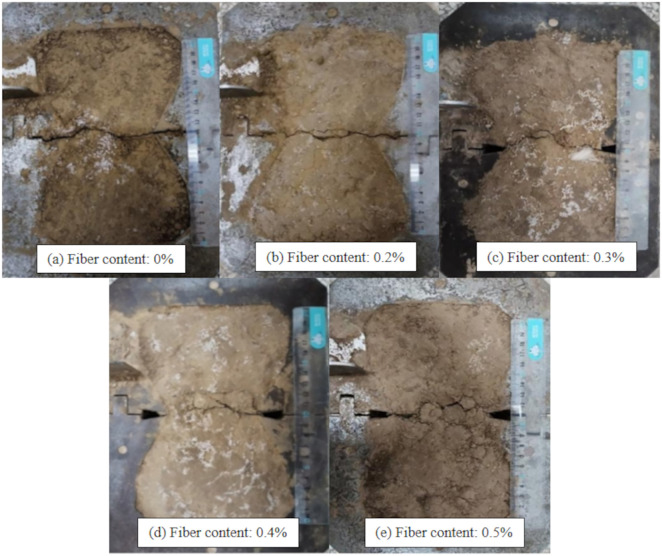
Planar fracture morphology of tensile test specimen.

**Fig 6:**
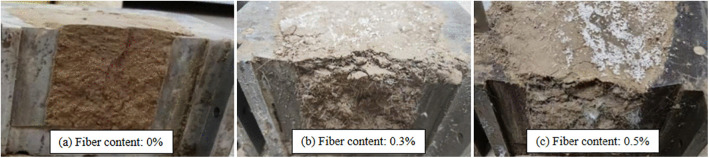
Fracture morphology of the cross-section of the tensile test specimen.

Frozen loess exhibits typical brittle fracture characteristics under tensile action, while the failure pattern of frozen loess modified by polypropylene fiber gradually changes from single brittle fracture to multi-crack and gradual failure. To investigate the tensile failure form of polypropylene fiber-reinforced loess, photographs shall be taken of the surface and cross-section form of the tensile specimens following completion of the tensile tests. Fig 5 shows the planar failure patterns of tensile tests conducted at a freezing temperature of −5°C, a loading rate of 1.57 mm/min, a moisture content of 16.2%, and a dry density of 1.80 g/cm³ for different fiber content ratios. Fig 6 shows its cross-sectional morphology diagram. As illustrated in Figs 5 and 6, the tensile test of frozen loess resulted in the formation of a single, broad, uninterrupted primary crack, characterised by a uniform and smooth fracture surface. The soil exhibited a single weak plane of separation, demonstrating characteristic brittle deformation. An increase in the polypropylene fiber content was observed to be concomitant with an increase in the number of main cracks, whilst the crack widths narrowed. Fine micro-crack branching occurs locally, forming a network of micro-cracks near the fracture surface. The fibers form effective restraint, with exposed fibers or pull-out marks visible at the fracture surface. Further increases in the polypropylene fiber content resulted in denser, more dispersed cracking, exhibiting ‘crazing’ or ‘multidirectional intersecting’ patterns. The fibers are dispersed to form a three-dimensional reinforcement network, which significantly inhibits crack propagation. The fracture surface exhibited unevenness, accompanied by extensive fiber exposure, resulting in a ‘fluffy’ morphology.

### 3.2. Stress-strain curve

**Fig 7:**
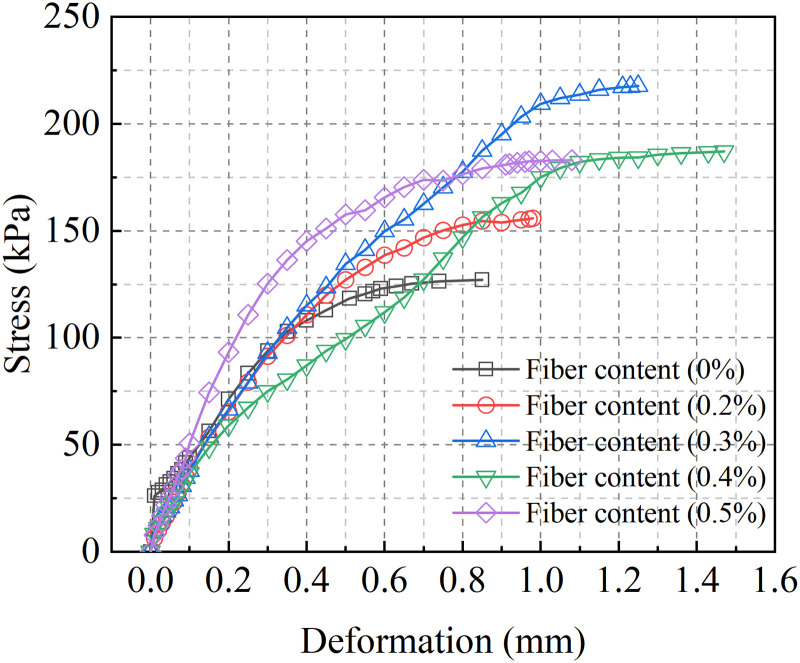
Stress-strain curves of frozen loess under conditions of −5°C and 1.57 mm/min for loess with varying fiber parameters.

The tensile stress-deformation curve of frozen loess and frozen improved loess is drawn from the stress-deformation data before the specimen is broken by tensile fracture. Fig 7 presents the stress-strain curves for frozen loess modified with different fiber parameters under a freezing temperature of −5°C and a loading rate of 1.57 mm/min. As evident from Fig 7, the incorporation of polypropylene fibers effectively enhances the tensile properties of the frozen soil, increasing both the peak stress and ductility of the specimens. At a freezing temperature of −5°C, the stress-strain curves of specimens with different fiber contents all exhibit a strain-hardening type. With increasing fiber content, the peak strength initially increases before decreasing; the maximum stress peak is observed at a fiber content of 0.3%. As can be seen from the cross-sectional view of the tensile specimen in Fig 6, when the fiber content is 0.3%, the fibers are distributed uniformly. At a fiber content of 0.5%, however, due to the abundance of fibers, some of them are found in bundles. A fiber parameter of 0.5% results in restricted fiber interaction within the bundle, with the interface between the bundle and frozen soil becoming a weak link, leading to stress concentration and initial defects. Which in turn reduces the peak strength of the specimen. Li et al. [45] came to the same conclusion: the influence of fiber parameters on the tensile strength of reinforced soil exhibits a critical threshold. Beyond this threshold, fiber dispersion deteriorates markedly, resulting in fiber agglomeration. During tensile deformation of the fiber-reinforced frozen loess, microcracks initiate under loading. The fiber network then bridges the crack surfaces and bears part of the tensile stress. This bridging action restricts crack opening and delays crack propagation. During this process, additional energy is dissipated through debonding at the fiber-frozen soil interface, fiber stretching, and fiber pullout, thereby significantly increasing the fracture energy of the specimen. Meanwhile, the presence of fibers improves the stress transfer pathways within the soil matrix. Consequently, local stress concentration is alleviated, and the initiation and propagation of the dominant crack are delayed. It is noteworthy that even at higher admixture levels (0.5%), where localized fiber agglomeration occurs, interactions within the fiber bundles and between them and the frozen soil continue to maintain bridging and energy dissipation, thereby sustaining the strain-hardening response.

**Fig 8:**
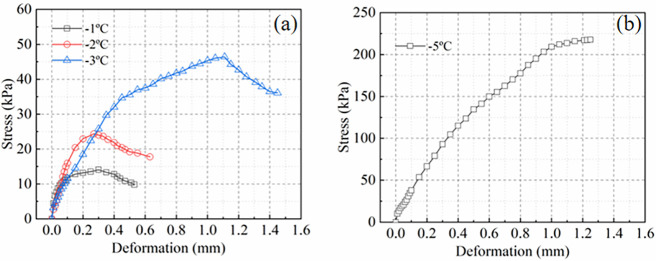
Stress-deformation curves of frozen modified loess with 0.3% fiber parameter at 1.57 mm/min under different temperature conditions.

Fig 8 presents the stress-strain curves of frozen-improved loess with 0.3% fiber content under varying temperature conditions at a loading rate of 1.57 mm/min. As shown in Fig 8, the peak force of the specimens increases with decreasing freezing temperature, and the stress-strain curve form of the improved loess transitions from strain-softening to strain-hardening. At freezing temperature, ice exhibits significant temperature dependence. As the freezing temperature decreases, the tensile strength of ice increases, whilst the content of unfrozen water within the specimen sharply diminishes. This enhances the cohesive action between soil particles themselves and between soil particles and fibers, thereby elevating the peak tensile strength of the modified loess. When the freezing temperature is high, the viscoelastic plasticity of improved frozen loess is strong. Initial microcracks propagate rapidly within weakly cemented zones, while fibers are extracted due to insufficient ice anchoring force, causing a sudden stress drop. Specimens exhibit a steep decline in the curve following the peak. As freezing temperatures decrease, the composite interaction between ice and fibers intensifies. Crack propagation must overcome the strong confinement of fibers, transitioning to a diffuse microcracking pattern. The specimen forms progressive strengthening, transforming into a strain-hardening form.

**Fig 9:**
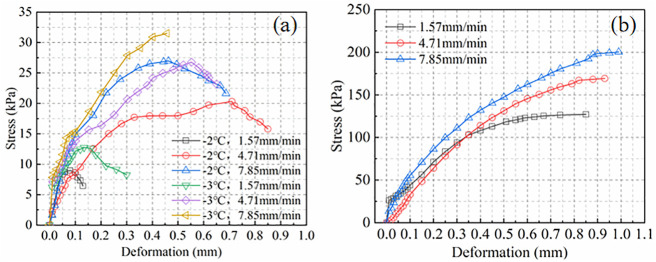
Stress-strain curves for loess frozen at different loading rates: (a) Freezing temperatures-of-2°C-and-3°C (b) Freezing temperature-of-5°C.

**Fig 10:**
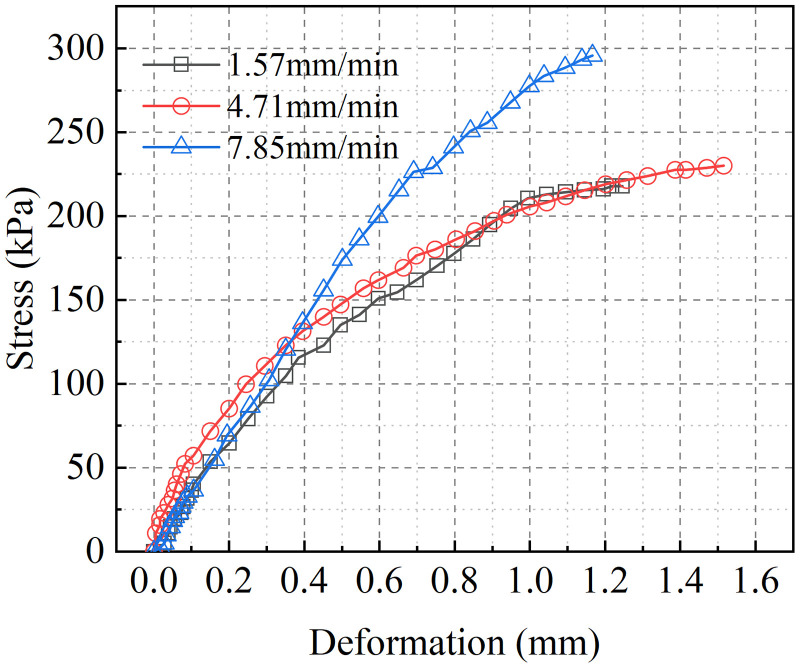
Stress-strain curves of freeze-improved loess with 0.3% fiber content at −5°C under different loading rates.

Figs 9 and 10 present the stress-strain curves for frozen loess and frozen modified loess under different loading rates. As shown in Figs 9 and 10, the peak stress of the specimens increases with the increase in loading rate. From the perspective of the stress-strain curve morphology, tensile tests on frozen loess at −2°C exhibit a strain-softening type. At −3°C, under the loading rate of 1.57 mm/min and 4.71 mm/min, it shows the strain softening type, and 7.85 mm/min shows the strain hardening type. Under the condition of −5°C, it shows a strain hardening type under the condition of each loading rate. The stress-strain curves of frozen-improved loess with 0.3% fiber content exhibited a strain hardening type across all loading rates. Analyse the reasons for the influence of loading rate on the stress-strain curve. Firstly, the ice within the frozen soil possesses viscoelastic-plastic behavior. During rapid loading, this manifests as heightened viscous resistance, leaving insufficient time for creep relaxation to occur, enhancing tensile strength. Moreover, as the loading rate increases, the migration and redistribution of unfrozen water are restricted, diminishing the lubricating effect of water. Consequently, the frictional resistance between soil particles intensifies, leading to an increase in the peak tensile stress. Zhang et al. [46] similarly confirmed that the peak stress of frozen ground increases significantly with increasing loading rate. Slow loading permits microcracks ample time to initiate at weak points, propagate steadily, and interconnect. This progressive damage accumulation leads to a reduction in the specimen’s load-bearing capacity during tensile testing, resulting in softening. Conversely, during rapid loading, microcracks lack time to propagate and coalesce after initiation. Consequently, the energy imparted by external loading is predominantly utilized for uniform elastic energy storage and limited plastic deformation, manifesting macroscopically as strain hardening of the specimen.

### 3.3. Tensile strength

**Fig 11:**
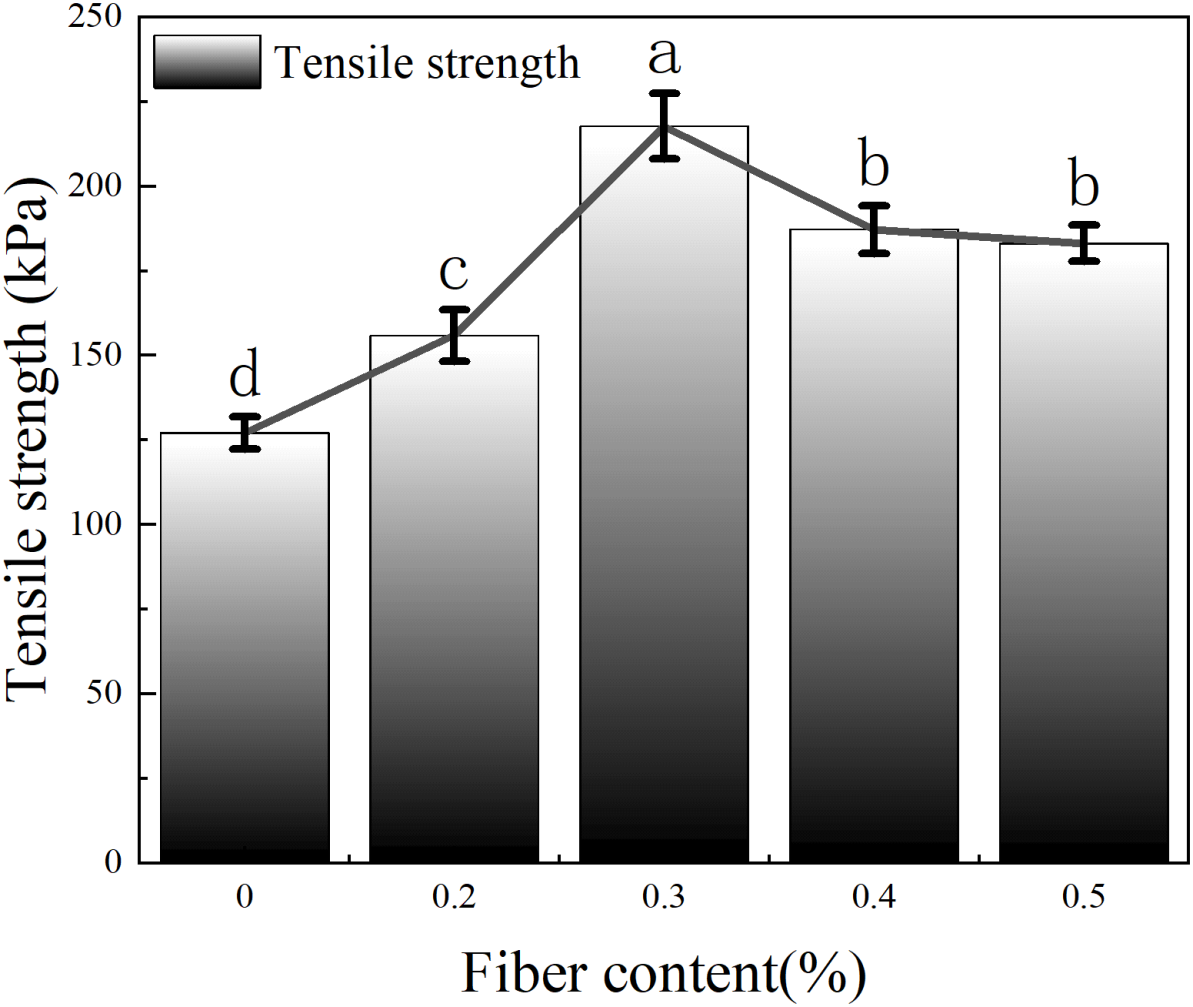
Effect of polypropylene fiber content on the tensile strength of frozen loess at −5°C and a loading rate of 1.57 mm/min. Different lowercase letters above the bars indicate statistically significant differences among groups according to Tukey’s HSD test (p < 0.05).

Since the addition of polypropylene fibers reduced the tensile brittleness of the frozen loess, the tensile strength of all specimens was determined as the peak strength on the stress-strain curve. Fig 11 shows the effect of polypropylene fiber content on the tensile strength of frozen loess at a freezing temperature of −5°C and a loading rate of 1.57 mm/min. One-way analysis of variance (ANOVA) indicated that fiber content had a highly significant effect on the peak stress of the specimens (F(4,10) = 68.71, P = 3.58 × 10 ⁻ ⁷). As indicated by the significance markers (a–d) in Fig 11 (significance order: a > b > c > d), Tukey’s post hoc test confirmed that the peak stress reached its absolute maximum (217.78 ± 9.70 kPa) when the fiber content was 0.3%. This value was significantly higher than that of all other experimental groups (P < 0.05). As shown in Fig 11, with increasing fiber content, the tensile strength of the modified loess initially increases rapidly. At a fiber content of 0.3%, the tensile strength reaches its peak, after which it gradually decreases with further increases in fiber content. This demonstrates that adding polypropylene fibers effectively enhances the frozen tensile strength of the specimens. The tensile strength at a fiber content of 0.3% is 71.4% greater than that of specimens without added fibers.

**Fig 12:**
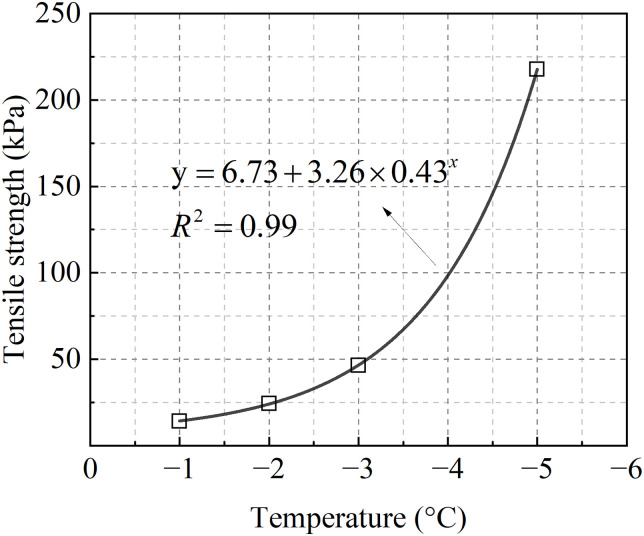
Effect of freezing temperature on the tensile strength of fiber-reinforced frozen loess at a loading rate of 1.57 mm/min and a fiber content of 0.3%. The exponential regression equation is $y=6.73+3.26\times {\text{0}.\text{43}}^{\text{x}}$, with $R^{\text{2}}=\text{0}.\text{99}$, where y is the tensile strength in kPa and x is the freezing temperature in ℃.

Fig 12 presents the influence of modified loess freezing temperature at a loading rate of 1.57 mm/min and 0.3% fiber content on the tensile strength of loess. Fig 12 demonstrates that the frozen tensile strength exhibits exponential growth as the freezing temperature decreases. This occurs because the reduction in temperature leads to an increase in the number of ice crystals and their strength index, and the thinning of the unfrozen water film diminishes the lubricating effect on soil particles, thereby enhancing the tensile strength of the specimen.

**Fig 13:**
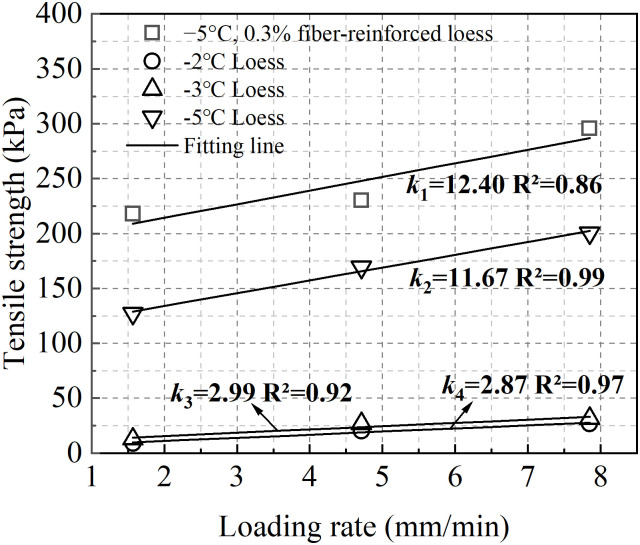
Relationship curve between tensile strength and loading rate. The fitted linear equations were y=189.5+12.4x, $R^{\text{2}}=\text{0}.\text{86}$ for the −5℃，0.3% fiber-reinforced loess; y=110.6+11.67x, $R^{\text{2}}=\text{0}.\text{99}$, for frozen loess at −5 ℃; y=9.6+2.99x, $R^{\text{2}}=\text{0}.\text{92}$, for frozen loess at −3 ℃; y=5.3+2.87x, $R^{\text{2}}=\text{0}.\text{97}$, for frozen loess at −2 ℃; where y is the tensile strength in kPa and x is the loading rate in mm/min.

Fig 13 shows the relationship curve between tensile strength and loading rate. From Fig 13, it can be observed that tensile strength exhibits a linear increase with increasing loading rate, which is the same rule as in reference [47]. As the freezing temperature decreases, the slope of the straight line is larger, indicating that the specimen becomes more sensitive to the loading rate. After adding polypropylene fibers, the slope of the straight line increases slightly, and the tensile strength is significantly greater than that without fiber addition. This demonstrates that adding fibers amplifies the effect of loading rate on tensile strength, which gives it greater advantages in engineering applications involving high-speed loading scenarios.

### 3.4. Mechanism of polypropylene fiber reinforcement

(1) **Qualitative Analysis of the Microstructure of Loess Reinforced with Polypropylene Fibers**

**Fig 14:**
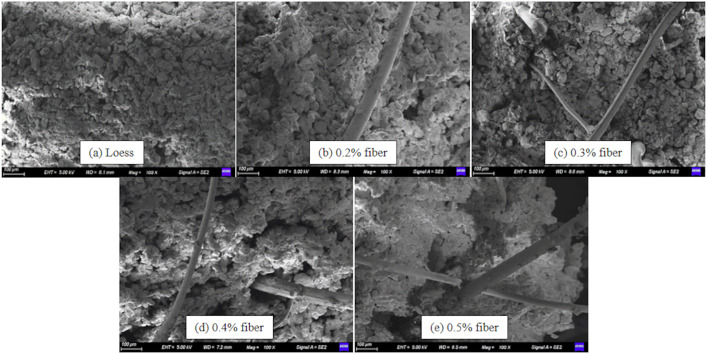
Microstructure SEM image (100×).

From the above analysis, it is known that 0.3% fiber content represents the optimal parameter for enhancing the tensile strength of frozen loess. Fig 14 shows SEM images of loess reinforced with varying fiber contents, where the part with greater grey scale is the soil pore and the part with lesser grey scale is the soil skeleton. From Fig 14(a), it can be observed that the frozen loess particles exhibit uniform distribution, primarily involving point-to-point or point-to-surface contact. The microporosity consists mainly of micro- and small-scale pores, with a relatively high porosity count; from Fig 14(b) to Fig 14(e), it can be observed that a single polypropylene fiber forms a favorable grip-wrap effect with the soil matrix. Multiple polypropylene fibers randomly distributed and interwoven within the soil skeleton create a fiber network, which enhances the tensile strength of the soil; when the frozen loess and frozen modified loess specimens were prepared, the filling quality of the soil samples remained consistent. So, Figs 14(b) and 14(c) demonstrate that increasing the fiber content enhances both face-to-face contact between loess particles and face-to-fiber contact, and the improved loess structure becomes compact when the fiber content is 0.3%. With the further increase of fiber parameters, the compaction effect of fiber and soil samples in local areas is not good due to the increase in fiber parameters. Coupled with some fibers being pulled out during tensile testing or preparation of SEM specimens, this resulted in an increase in the number of large pores within the specimens.

(2) **Quantitative Analysis of the Microstructure of Polypropylene Fiber-Reinforced Loess**

**Fig 15:**
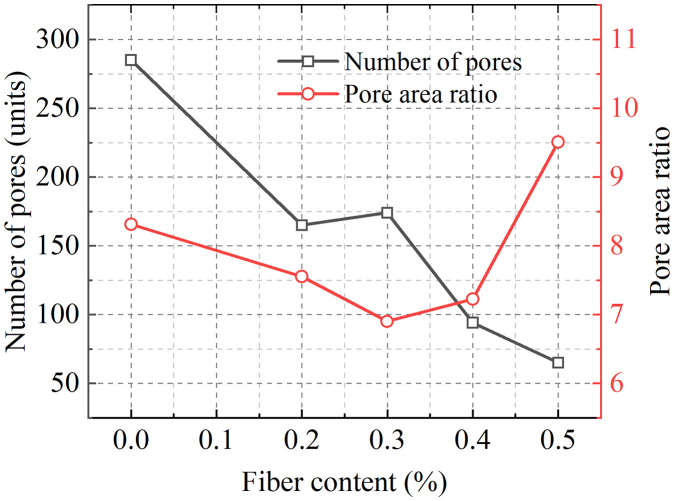
Effect of Fiber Parameters on Pore Number and Proportion of Pore Area.

**Fig 16:**
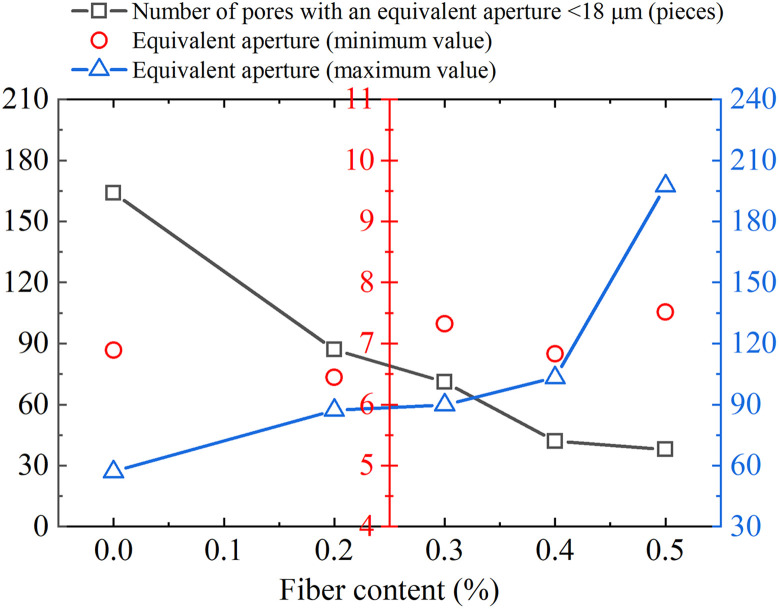
Equivalent Aperture Microscopic Parameters.

Using Image-Pro Plus 6.0 image analysis software, microscopic pore structure parameters were extracted from SEM images to analyze, at the microscopic level, the mechanism by which polypropylene fibers reinforce the frost resistance of frozen loess. Fig 15 depicts the variation curves of pore count and pore area ratio (ratio of pore area to total area) with respect to fiber parameters. From Fig. 15, the pore count exhibits an overall decreasing trend with increasing fiber parameters, and the pore area ratio shows a decrease followed by an increase. If irregular pores are approximated as circular, then the pore area can be converted to an equivalent diameter characterization, with Fig 16 presenting the microscopic parameter diagram for equivalent pore size. Fig 16 indicates that the minimum equivalent aperture across different parameter ranges is approximately from 6.5 to 7.5 μm. The maximum equivalent aperture exhibits an increasing trend with rising fiber parameters, whilst the number of small pores smaller than 15 μm diminishes as fiber parameters increase. Analysis of the reasons for the above micro-parameters reveals that small and micro-voids are filled by fibers and a denser soil skeleton. Even at high parameters, fibers continue to fill small voids; however, this filling effect is counterbalanced by the simultaneous generation of large voids, with an overall trend towards reduced void quantity. At parameter quantities less than or equal to 0.3%, the effective filling of fibers and mesh reinforcement results in the overall densification of the specimen structure. Small voids are filled, large voids are rare, and the void area is reduced; when the parameter exceeds 0.3%, although the small voids continue to diminish, the proportion of large voids formed by fibers being pulled out increases due to inadequate compaction caused by fiber agglomeration. The increment in newly formed macropore area significantly exceeded the reduction in micropore area, resulting in an overall increase in the proportion of total pore area and surpassing that in the case of unmixed loess.

Therefore, achieving the appropriate fiber content (0.3%) fundamentally depends on approaching the microstructural equilibrium point. At this critical threshold, the fibers fully exert their role in filling small pores and consolidating the soil, maximizing structural integrity before the increase in macropore area caused by aggregation overrides the filling effect. When fiber content becomes excessive, the positive effect of fibers filling small pores persists, yet the number of pores continues to decrease. However, it is obscured by the negative effects of poor compaction caused by fiber agglomeration and large holes formed by fiber pull-out. The adverse effects significantly increased the area of large pores, leading to a rise in the proportion of total pore area. Consequently, the soil structure became loose, manifesting macroscopically as a reduction in tensile properties.

## 4. Conclusion

(1) Tensile testing of frozen loess and frozen loess modified with polypropylene fibers both exhibited brittle fracture failure, though the failure modes differed. The tensile cracks in frozen loess were wide and continuous, with a flat fracture surface. Frozen fibers in modified loess produce numerous tensile cracks, forming a network of microcracks near the fracture surface; the fracture face reveals exposed fibers or pulled-out traces. Particularly at higher fiber content levels, cracks exhibit a ‘crackling’ or ‘multidirectional intersecting’ pattern, with fracture surfaces becoming uneven. The large number of exposed fibers presents a ‘fuzzy’ appearance on the cross-section.
(2) The tensile properties of frozen loess and its polypropylene fiber-modified soil are significantly affected by fiber content, freezing temperature, and loading rate. The incorporation of polypropylene fibers effectively enhances the tensile strength of frozen modified soil. The strength exhibits an initial increase followed by a decrease as the fiber content rises (with 0.3% being optimal). At a 0.3% fiber content, the strength surpasses that of frozen loess by 71.4%. The tensile strength of both types of soil increases with the decrease of freezing temperature, and the shape of the stress-deformation curve changes from the strain-softening type to the strain-hardening type. In addition, the tensile strength of both increases linearly with the increase in loading rate.
(3) Incorporating polypropylene fibers into loess effectively inhibits the development of micro- and small-scale pores during freezing. The fibers form a favorable grip-wrap effect with the soil matrix, and their randomly distributed network structure significantly enhances the soil’s tensile strength. However, excessive fiber content leads to an increase in large pores and raised porosity, resulting in a loosening of the soil structure and consequently weakening its tensile strength.
