# Supplementary material for: Study on tensile mechanical response and microstructure of polypropylene fiber reinforced loess under freezing
Source: PLoS One. 2026 Jun 9;21(6):e0350932. doi: 10.1371/journal.pone.0350932 (PMC13249173; doi:10.1371/journal.pone.0350932)
Supplement: Attachment — Submitted filename: Response to reviewers.docx [file pone.0350932.s002.docx]

Dear Editors and Reviewers:

Thank you for your letter and for the reviewers comments concerning our manuscript entitled“**Study on tensile mechanical response and microstructure of polypropylene fiber reinforced loess under freezing**”(ID: **PONE-D-26-00128**). Those comments are all valuable and very helpful for revising and improving our paper, as well as the important guiding significance to our researches. According to your nice suggestions, we have made extensive corrections to our previous draft. We have added necessary data to supplement our results and edited our article extensively. The detailed corrections are listed below.

Responds to the academic editor and reviewer's comments:

Academic editor's requirements:

1. Please ensure that your manuscript meets PLOS ONE's style requirements, including those for file naming.

**Response:** Based on the downloaded template files, we have revised the article to ensure meet PLOS ONE's style requirements.

2. In your Methods section, please provide additional information regarding the permits you obtained for the work.Please ensure you have included the full name of the authority that approved the field site access and, if no permits were required, a brief statement explaining why.

**Response:** Thank you for your comment. We would like to clarify that no permit was required for this study.

The soil used in this study was collected from Daqingshan in Anning District, Lanzhou, China. This site is a non-protected public area and does not involve any endangered or protected species. Therefore, no specific fieldwork or collection permits were required for this study. Regarding the details of the soil sampling area, we have made relevant revisions in Section 2.2 ‘Test Soils and Sample Preparation’ of the paper; please refer to lines 160–162.

a) **The revised content:** The soil used in this study was collected from Daqingshan in Anning District, Lanzhou, China, at a depth of 0.5–2.0 m below the surface, and belongs to the Quaternary Lanzhou Loess.

b) **The original content:** The experimental soil consists of Quaternary Lanzhou Loess buried at a depth of 0.5–2.0 m.

3. Please note that PLOS One has specific guidelines on code sharing for submissions in which author-generated code underpins the findings in the manuscript. In these cases, we expect all author-generated code to be made available without restrictions upon publication of the work.

**Response:** Thank you for the reminder regarding PLOS One’s code sharing policy. We would like to clarify that this study did not involve any author-generated code or specialized software scripts to underpin the findings. All data analysis and statistical tests (e.g., ANOVA, plotting in Origin) were performed using standard, commercially available software. Therefore, the code-sharing requirement is not applicable to this manuscript.

4. We note that your Data Availability Statement is currently as follows: “All relevant data are within the manuscript and its Supporting Information files.”Please confirm at this time whether or not your submission contains all raw data required to replicate the results of your study. Authors must share the “minimal data set” for their submission. PLOS defines the minimal data set to consist of the data required to replicate all study findings reported in the article, as well as related metadata and methods

**Response:** We apologize that we did not specify where to find the minimal dataset on which our experimental results are based. We have uploaded the data into an online database, which can be found at the link below.

<https://doi.org/10.6084/m9.figshare.31812757>

5. Please upload a copy of Supporting Information which you refer to in your text on page 27-28

**Response:** Thank you for pointing this out. We apologize for the oversight in the initial submission. We have now uploaded the Supporting Information file to the submission system. This file contains all the additional data and figures referenced on pages 27-28 of the manuscript.

6. We note that Figures 1, 3, 4 and 12 in your submission contain copyrighted images. All PLOS content is published under the Creative Commons Attribution License (CC BY 4.0), which means that the manuscript, images, and Supporting Information files will be freely available online, and any third party is permitted to access, download, copy, distribute, and use these materials in any way, even commercially, with proper attribution. For more information, see our copyright guidelines: <http://journals.plos.org/plosone/s/licenses-and-copyright.>

**Response:** Thank you for the reminder regarding copyright. We would like to clarify that Figures 1, 3, 4, and 12 are original works created by the authors of this study.

7. If the reviewer comments include a recommendation to cite specific previously published works, please review and evaluate these publications to determine whether they are relevant and should be cited. There is no requirement to cite these works unless the editor has indicated otherwise.

**Response:** We have noted the policy regarding the citation of previously published works. We confirm that all citations in the revised manuscript have been evaluated for their relevance and necessity to support our findings.

**Reviewer #1:**

1. **Comment:** In line 241, is the failure modes of test specimens showing the same brittle fracture failure at different moisture content?

**Response:** We sincerely thank the reviewer for this insightful comment. First, we would like to emphasize that in our experimental design, the moisture content of all samples was strictly controlled as a constant variable at 16.2% (see Table 1 for details). Therefore, the change in failure mode is not driven by moisture content. However, we fully agree with your core criticism that describing all failure modes as “brittle fracture” is inaccurate. In the revised manuscript, we have accurately described how the fracture mode transitions from quasi-brittle to ductile behavior upon fiber incorporation, as detailed in lines 348–350.

a) **The revised contents:** Since the addition of polypropylene fibers reduced the tensile brittleness of the frozen loess, the tensile strength of all specimens was determined as the peak strength on the stress-strain curve.
b) **The origin contents:** As the tensile specimens all exhibited brittle fracture failure, the tensile strength of the specimen was taken as the peak stress value from the stress-strain curve.
2. **Comment:** Please providing further information about the test specimen, such as dimensions, shapes or etc

**Response:** We greatly appreciate your careful review and valuable feedback. We fully agree that the specific geometric details of the test specimens are crucial for understanding stress distribution and ensuring the reproducibility of the experiment. We sincerely apologize for the omission of these details in the original manuscript. We have now explicitly included these details regarding the shape and dimensions of the specimens in the revised manuscript, as described in lines 120–158.

**a) The revised contents:** The overall dimensions of the tensile box are 300 mm × 200 mm × 105 mm. The internal chamber of the tensile box forms a butterfly-shaped tensile interface through geometric contraction, with a total volume of 1840 cm³. The geometric dimensions of the specimen housed within the box are 225 mm × 145 mm × 75 mm, as shown in Figure 2. During the tensile process, the critical tensile longitudinal cross-section of the specimen (i.e., the butterfly-shaped contact surface) measures 75mm × 75mm.

（a）	（b）

**Fig. 2 (a) Overall design of the tensile testing box and (b) schematic diagram (units: mm).**

**b) The origin contents:** The interior of the tensile chamber is configured in the form of a bow-tie design, with a chamber volume of 1840 cm³. The specimen under tension has longitudinal cross-sectional dimensions of 75 mm × 75 mm.

3. **Comment:** In fig 6, the stress-deformation curve of the soil sample under-5℃ does not work as a kind of brittle material, this may be contradictory to the conclusion in line 241.

**Response:** We greatly appreciate your careful review and valuable suggestions. The stress-strain curves below -5°C (see Figure 6) clearly exhibit sustained deformation and strain hardening/softening characteristics, indicating that the material exhibits a ductile response rather than purely brittle behavior. The original vague statement on line 241 was indeed a general error that contradicted our data. We have corrected this in the revised manuscript (lines 348–350), highlighting the ductile transition to ensure strict consistency between the textual description and the experimental curves.

**a) The revised contents:** Since the addition of polypropylene fibers reduced the tensile brittleness of the frozen loess, the tensile strength of all specimens was determined as the peak strength on the stress-strain curve.

**b) The origin contents:** As the tensile specimens all exhibited brittle fracture failure, the tensile strength of the specimen was taken as the peak stress value from the stress-strain curve.

4. **Comment:** There is also the same doubt in Fig 7

**Response:** We thank the reviewers for their keen observations. Similar to the case in Figure 6, the stress-strain curves in Figure 7 under different loading rates also exhibit varying degrees of ductility and post-peak deformation. It is inaccurate to collectively refer to this phenomenon as “brittle fracture.” The original, vague statement in line 241 was indeed a error that contradicted our data. We have corrected this in the revised manuscript (lines 348–350), highlighting the ductility transition to ensure strict consistency between the textual description and the experimental curves.

**a) The revised contents:** Since the addition of polypropylene fibers reduced the tensile brittleness of the frozen loess, the tensile strength of all specimens was determined as the peak strength on the stress-strain curve.

**b) The origin contents:** As the tensile specimens all exhibited brittle fracture failure, the tensile strength of the specimen was taken as the peak stress value from the stress-strain curve.

5. **Comment:** How to get an appropriate amount of fiber fully motivating the role in filling small pores and fiber reinforcing densification?

**Response:** We sincerely thank the reviewers for raising these insightful and critical questions regarding the reinforcement mechanism. In our study, the “optimal amount” was determined by identifying a microstructural equilibrium point—specifically, the critical threshold between the formation of an effective spatial network and the onset of fiber aggregation. As discussed in Section 3.4 regarding the analysis of the reinforcement mechanism for polypropylene fibers: when the fiber content is below the optimal level, the fibers are too sparse to form an interconnected network, resulting in limited pore-filling effects. When the fiber content reaches the appropriate level (0.3%), the fibers are uniformly distributed and cross-overlap, effectively partitioning large pores and filling micropores. At this specific threshold, “fiber-reinforced compaction” is fully activated. Once the fiber content becomes too high (e.g., 0.4% or 0.5%), the limited spatial distance causes the fibers to become entangled and agglomerate. This agglomeration effect prevents soil particles from integrating into the network and creates large voids when the fibers are pulled out, ultimately weakening the structure. We have revised lines 448–452 to fully incorporate an explanation of this quantitative mechanism into the revised draft.

**a) The revised contents:** Therefore, achieving the appropriate fiber content (0.3%) fundamentally depends on approaching the microstructural equilibrium point. At this critical threshold, the fibers fully exert their role in filling small pores and consolidating the soil, maximizing structural integrity before the increase in macropore area caused by aggregation overrides the filling effect.

**b) The origin contents:** Therefore, an appropriate amount of fiber can fully leverage its role in filling small pores and fiber-reinforced densification while simultaneously reducing the number of pores and their proportion of the total pore area. The optimal microstructure is obtained, corresponding to the best tensile properties.

**Reviewer #2:**

1.**Comment:** The manuscript does not clearly indicate the number of specimens tested for each condition. Please report the number of replicates and include measures of variability such as standard deviation or error bars. Without this information, it is difficult to assess the reliability of the reported results.

**Response:** We greatly appreciate the reviewers’ rigorous comments regarding the reliability of our experiments. We sincerely apologize for failing to clearly explain the experimental replication process in the original manuscript.

During the experimental phase, we strictly adhered to the requirements for replication, conducting three parallel experiments (N=3) for each of the experimental conditions listed in Table 1 to ensure the reliability of the data. Furthermore, we performed a comprehensive statistical analysis of the replicate groups; the deviation between the single-test strength values and the average strength values from the three replicate tests did not exceed 8% under any experimental condition. The relative error between replicate samples was minimal, and the presented mean values accurately reflect the true trend of the parameters. To maintain the visual clarity of the complex stress–strain curves and avoid data redundancy, we have chosen to present the mean values in the figures.

Translated with DeepL.com (free version)To directly address your concerns and enhance the rigor of the manuscript, we have explicitly outlined the experimental replication procedure in lines 195–199.

**a) The revised contents:** The test conditions are shown in Table 1. For each test condition, three replicate tests (N=3) were conducted, and the deviation between the individual strength values and the average strength value for the three replicates should not exceed 8% to ensure the reliability of the data. The data shown in the figure represent the average strength values from the replicate tests.

2. **Comment:** The conclusion that 0.3% fiber content is optimal should be supported by statistical analysis. Please provide appropriate statistical tests (e.g., ANOVA or similar) to demonstrate that the differences between fiber contents are significant.

**Response:** We sincerely thank the reviewers for their rigorous and constructive suggestions. We fully agree that statistical analysis is crucial for validating our conclusions regarding the optimal fiber content.

Following your recommendation, we conducted a one-way analysis of variance (ANOVA) and Tukey’s post-hoc test to comprehensively assess the significance of differences in peak stress among the composite groups. The statistical results (F(4,10) = 68.71) indicate that fiber content has a significant effect on peak stress. Specifically, Tukey’s post-hoc analysis confirmed that the peak stress in the 0.3% group was significantly higher than in all other experimental groups (P < 0.05 for all pairwise comparisons with the 0.3% group). This provides strong statistical evidence that 0.3% is indeed the optimal fiber content.

We have updated the manuscript on lines 352–384 to incorporate these statistical findings. We have also revised Figure 11, adding standard error bars and statistical significance labels (a–d) to visually convey these results.

**a) The revised contents:** One-way analysis of variance (ANOVA) indicated that fiber content had a highly significant effect on the peak stress of the specimens (F(4,10) = 68.71, P = 3.58 × 10⁻⁷). As indicated by the significance markers (a–d) in Figure 11 (significance order: a > b > c > d), Tukey’s post hoc test confirmed that the peak stress reached its absolute maximum (217.78 ± 9.70 kPa) when the fiber content was 0.3%. This value was significantly higher than that of all other experimental groups (P < 0.05).

**Fig. 11** **Effect of Polypropylene Fiber Parameters on the Tensile Strength of Frozen Loess at -5°C and 1.57 mm/min.**

3. **Comment:** The novelty of the study should be clarified. Similar trends regarding fiber dosage effects, temperature dependence, and loading rate influence have been reported in previous studies. The authors should clearly state what new contribution this work provides.

**Response:** We sincerely thank this reviewer for their critical and thought-provoking comments. We agree that the macroscopic trends observed in our study—such as the increase in strength at the optimal fiber dosage and the temperature-dependent strengthening effect—are highly consistent with the existing literature. In fact, this consistency further validates the reliability and accuracy of our experimental testing system. However, we respectfully emphasize that the primary novelty and core contribution of this work extend far beyond these macroscopic trends.

① It focuses on the tensile strength of fiber-reinforced frozen loess—a specific material system relevant to engineering in cold regions that has previously received insufficient attention.

② A custom-designed direct tensile testing apparatus was employed, thereby overcoming the limitations of the indirect testing methods commonly used in previous studies.

③ This study systematically examined the interactions among fiber content, freezing temperature, and loading rate, revealing how these factors influence one another and, in turn, affect mechanical properties.

④ The study provides microstructural evidence explaining the transition from brittle to ductile fracture and the “optimal fiber content” phenomenon (i.e., 0.3% fiber content)—at this level, appropriate fiber incorporation fills voids and forms a reinforcing network, whereas excessive fiber leads to agglomeration and weakening.

To better highlight the innovation and contributions of this study, we have revised the Introduction (lines 100–109).

**a) The revised contents:** Furthermore, existing experimental equipment for frozen soil tensile testing often faces challenges in securing specimens, preventing localized damage, and accommodating fiber-frozen soil composites. These limitations hinder the accurate characterization of the tensile failure mechanisms and mechanical responses of fiber-reinforced soils in frozen environments. To address these issues, this study independently designed and developed a dedicated frozen soil tensile testing apparatus that ensures effective specimen clamping and minimizes stress concentration during loading. Using this apparatus, a systematic series of tensile tests was conducted on polypropylene fiber-modified loess under frozen conditions.

**b) The origin contents:** Concurrently, existing equipment or methodologies for investigating the tensile properties of randomly distributed fiber-modified loess in frozen states remain limited in ensuring effective specimen clamping, avoiding localised damage, and being suitable for fiber-frozen soil composites. To more accurately capture the mechanical response and failure characteristics of such modified frozen soils under tensile loading, this study independently designed and developed a specialised frozen soil tensile testing apparatus. Employing this apparatus, systematic tensile tests were conducted on polypropylene fiber-reinforced loess under frozen conditions.

4. **Comment:** The self-developed tensile testing device is an important part of the study, but its accuracy and reliability are not discussed. Please provide information on calibration, validation, or comparison with existing methods.

**Response:** We sincerely thank this reviewer for their rigorous and highly professional comments. We fully agree that validating the in-house developed equipment is a key prerequisite for ensuring the credibility of the entire study. We conducted validation of the experimental data immediately after the tensile tester was completed. To directly address your concerns and clearly demonstrate the reliability of the equipment, we have now included details of the equipment validation in the revised manuscript. Specifically, we would like to clarify the following:

① Calibration: All core measurement components (such as high-precision load cells and displacement sensors) undergo rigorous factory calibration and are zeroed before each test to ensure sensor accuracy.

② Comparison with Existing Methods: Most importantly, during the preliminary research phase, we conducted systematic comparative validation using the classic split-tensile test as a benchmark. The direct tensile strength data obtained from our in-house developed equipment yielded a ratio of 0.86 compared to the results from the classic split tensile test, which is consistent with existing research findings (Perras et al. found that the strength from direct tensile tests is typically only 70%–90% of that from the classic Brazilian split test, doi:10.1007/s10706-014-9732-0). This cross-validation effectively demonstrates the mechanical accuracy of our equipment.

We have added a dedicated section in the experimental design section (lines 201–227) to provide a detailed explanation of this comparative validation and the reliability of the equipment, in order to maintain transparency for the reader.

**a) The revised contents:**

**2.3 Equipment Validation**

To verify the accuracy of the self-developed tensile testing machine, comparative experiments were conducted in this study. The reliability of the device was evaluated by comparing the results obtained from the tensile testing machine with those from the classic Brazilian splitting test. Under room temperature conditions (20°C), three sets of parallel experiments were conducted using the two methods described above to measure the tensile strength of loess samples (moisture content 16.2%, dry density 1.8 g/cm³). The data shown in the figure represent the average strength values from the parallel experiments. As shown in Figure 4, the peak tensile strength measured by the self-developed tensile testing machine was 15.12 kPa, while the peak strength measured by the classic Brazilian splitting test method was 17.58 kPa; the former was 86% of the latter. The relationship between the data from the uniaxial tensile test in this study and the classic Brazilian splitting test is similar to the results of previous studies [43,44].This is because, in the classic Brazilian splitting test, the combined tensile-compressive stress state within the specimen and the end-face friction effect inhibit the free propagation of microcracks, whereas the self-developed tensile testing machine performs uniaxial tensile testing, reducing the influence of compressive stress; macroscopic fracture always initiates first at localized microdefects or large voids within the specimen. Therefore, due to the combined influence of composite tensile-compressive stress constraints and end-face friction boundary effects, results obtained from the classic Brazilian split test are often higher than those from uniaxial tensile testing. This confirms that the self-developed tensile testing apparatus can effectively avoid interference from boundary compression effects and is a reliable method for obtaining true uniaxial tensile strength parameters of loess.

**Fig. 4 Stress-strain curves of loess under different testing methods**

References：

43.	Packulak TRM, Day JJ, McDonald MR, Jacksteit AC, Diederichs MS. Measurement of true tensile strength from Brazilian tensile strength laboratory tests. Canadian Geotechnical Journal. 2025;62: 1–14. doi:10.1139/cgj-2023-0204

44.	Perras MA, Diederichs MS. A Review of the Tensile Strength of Rock: Concepts and Testing. Geotechnical and Geological Engineering. 2014;32: 525–546. doi:10.1007/s10706-014-9732-0

The experimental procedure requires more detail, particularly regarding specimen preparation, fiber mixing and dispersion, freezing conditions (freezing duration and temperature control), and measures taken to ensure uniformity.

**Response:** We sincerely thank the reviewers for pointing out this important omission. We agree that a detailed description of the experimental procedures is essential for the reproducibility and reliability of the study. We have added further details to the section “2.2 Soil Samples and Sample Preparation.” Please refer to lines 179–187 for the relevant information.

**a) The revised contents:** Manually untangle the bundled fibers, then gradually mix them with dry soil using a mechanical mixer for 5 minutes. Next, add water to achieve the predetermined moisture content and mix thoroughly for 5 minutes, followed by allowing the soil to stand under sealed conditions for 12 hours.

For constant-temperature freezing, Place the tensile test specimen in a low-temperature chamber pre-cooled to the target temperature and maintain it at that temperature for 24 hours.

**b) The origin contents:** The fiber bundles were disentangled, and individual filaments were uniformly mixed into the dry soil. Water was added according to the predetermined moisture content, and the mixture was thoroughly mixed. The soil was then sealed and allowed to rest for 12 hours under controlled conditions.

For constant-temperature freezing, place the tensile box in a cryogenic chamber that has been pre-cooled to the target temperature.

5. **Comment:** The statements that tensile strength varies exponentially with temperature and linearly with loading rate should be supported by regression equations and goodness-of-fit values.

**Response:** We are very grateful to the reviewers for their constructive and thorough feedback. We fully agree that providing precise mathematical models and their goodness-of-fit statistics significantly enhances the quantitative credibility of our conclusions. Following your suggestion, we have added the linear regression equations and included the goodness-of-fit values in Figures 12 and 13 on lines 385–389 of the revised manuscript.

**a) The revised contents:**

**Fig. 12 Effect of modified loess freezing temperature at a loading rate of 1.57 mm/min and 0.3% fiber content on loess tensile strength.**

**Fig. 13 Relationship curve between tensile strength and loading rate.**

6. **Comment:** The SEM analysis is mainly descriptive. If possible, please include quantitative information or provide a clearer explanation of how the observed microstructure supports the mechanical results.

**Response:** We sincerely thank the reviewers for their constructive suggestions. We fully agree that explicitly linking microstructural observations to macroscopic mechanical behavior is crucial to the scientific depth of this study.

We would like to clarify that our study does indeed include a quantitative analysis of SEM images. As described in Section 3.4 (Quantitative Analysis of the Microstructure of Polypropylene Fiber-Reinforced Loess) (lines 486–529), we used Image-Pro Plus software to extract parameters of the soil’s microporous structure from SEM images, thereby analyzing the mechanism underlying the influence of polypropylene fiber reinforcement on the tensile properties of frozen loess from a microscopic perspective.

The analysis indicates that the number of pores generally decreases with increasing fiber content, while the proportion of pore area first decreases and then increases. The minimum equivalent pore diameter for different fiber contents is approximately 6.5–7.5 μm; the maximum equivalent pore diameter increases with increasing fiber content, and the number of small pores (less than 15 μm) decreases as fiber content increases. The reason driving these changes in microscopic parameters is that small and micro-pores are filled by fibers and a denser soil matrix. Even at high fiber content, fibers continue to fill small pores; however, this filling effect is offset by the large pores formed simultaneously, resulting in an overall trend of decreasing pore count. We have revised lines 448–452 to fully incorporate an explanation of this quantitative mechanism into the revised draft.

a) **The revised contents:** Therefore, achieving the appropriate fiber content (0.3%) fundamentally depends on approaching the microstructural equilibrium point. At this critical threshold, the fibers fully exert their role in filling small pores and consolidating the soil, maximizing structural integrity before the increase in macropore area caused by aggregation overrides the filling effect.

b) **The origin contents:** Therefore, an appropriate amount of fiber can fully leverage its role in filling small pores and fiber-reinforced densification while simultaneously reducing the number of pores and their proportion of the total pore area. The optimal microstructure is obtained, corresponding to the best tensile properties.

7.**Comment:** Raw experimental data (e.g., stress–strain curves or numerical values) should be provided as Supporting Information in accordance with data availability requirement.

**Response:** We greatly appreciate the reviewers’ suggestions and fully agree on the importance of data transparency and reproducibility in scientific research. To strictly comply with data availability requirements and provide robust support for our findings, we have compiled the raw experimental data into an Excel spreadsheet and uploaded it to the Figshare repository.

https://doi.org/10.6084/m9.figshare.31812757
