# Supplementary material for: Study on tensile mechanical response and microstructure of polypropylene fiber reinforced loess under freezing
Source: PLoS One. 2026 Jun 9;21(6):e0350932. doi: 10.1371/journal.pone.0350932 (PMC13249173; doi:10.1371/journal.pone.0350932)
Supplement: Attachment — Submitted filename: Response_to_reviewers_auresp_2.docx [file pone.0350932.s003.docx]

Dear Editors and Reviewers:

Thank you for your letter and for the reviewers comments concerning our manuscript entitled“**Study on tensile mechanical response and microstructure of polypropylene fiber reinforced loess under freezing**”(ID: **PONE-D-26-00128**). Those comments are all valuable and very helpful for revising and improving our paper, as well as the important guiding significance to our researches. According to your nice suggestions, we have made extensive corrections to our previous draft. The detailed corrections are listed below.

Responds to the reviewer's comments

**Reviewer #2:**

1.**Comment:** In Section 3.1 Please revise line 232‑233 to clearly distinguish the brittle failure of plain frozen loess from the ductile, multiple‑cracking failure of fiber‑reinforced specimens.

**Response:** Thank you for your valuable suggestion. We agree that the distinction between the failure modes of plain frozen loess and fiber-reinforced specimens should be stated more clearly. Accordingly, we have revised Section 3.1, Lines 230–232, to clarify that the plain frozen loess specimens exhibited typical brittle failure, characterized by abrupt tensile fracture and the formation of a dominant main crack. In contrast, the fiber-reinforced specimens showed a more ductile and progressive multiple-cracking failure pattern due to the crack-bridging and pull-out effects of polypropylene fibers. The corresponding discussion has been added in the revised.

**a) The revised contents: Frozen loess exhibits typical brittle fracture characteristics under tensile action, while the failure pattern of frozen loess modified by polypropylene fiber gradually changes from single brittle fracture to multi-crack and gradual failure.**

**b) The origin contents: The tensile test failure of frozen loess and frozen polypropylene fiber-modified loess is brittle fracture failure.**

2.**Comment:** Please replace the current Data Availability Statement with the Figshare DOI and confirm that the data are publicly accessible.

**Response:** Thank you for your helpful comment. We have revised the Data Availability Statement accordingly. The data supporting the findings of this study have been deposited in Figshare and are publicly accessible at https://doi.org/10.6084/m9.figshare.31812757. We have confirmed that the dataset is publicly accessible. This revision has been made in the revised manuscript, Lines 505–506.

**a) The revised contents: The data supporting the findings of this study have been deposited in Figshare and are publicly accessible at https://doi.org/10.6084/m9.figshare.31812757.**

**b) The origin contents: Data will be made available on request.**

3.**Comment:** Please explicitly write the exponential regression equation (with constants and R²) for Figure 12, and the linear equation (slope, intercept, R²) for Figure 13 in the respective figure captions or in the Results section (lines 385‑389). This is essential for quantitative reproducibility.

**Response:** Thank you for your valuable input. We agree that explicitly proposing regression equations is important for quantitative reproducibility. In line 390-399, we have modified the annotation of Figure 12 and Figure 13 to explicitly write the regression equation (with constant terms and R²).

**a) The revised contents: Fig. 12. Effect of freezing temperature on the tensile strength of fiber-reinforced frozen loess at a loading rate of 1.57 mm/min and a fiber content of 0.3%.The exponential regression equation is** $\text{y=6.73+3.26×}\text{0.43}^{\text{x}}$**, with** $\text{R}^{\text{2}}\text{=0.99}$**, where y is the tensile strength in kPa and x is the freezing temperature in ℃.**

**Fig. 13 Relationship curve between tensile strength and loading rate.The fitted linear equations were** $\text{y=189.5+12.4x}$**,** $\text{R}^{\text{2}}\text{=0.86}$ **for the -5℃，0.3% fiber-reinforced loess;** $\text{y=110.6+11.67x}$**,** $\text{R}^{\text{2}}\text{=0.99}$**, for frozen loess at -5 ℃;** $\text{y=9.6+2.99x}$**,** $\text{R}^{\text{2}}\text{=0.92}$**, for frozen loess at -3 ℃;** $\text{y=5.3+2.87x}$**,** $\text{R}^{\text{2}}\text{=0.97}$**, for frozen loess at -2 ℃; where y is the tensile strength in kPa and x is the loading rate in mm/min.**

**b) The origin contents: Fig. 12 Effect of modified loess freezing temperature at a loading rate of 1.57 mm/min and 0.3% fiber content on loess tensile strength.**

**Fig. 13 Relationship curve between tensile strength and loading rate.**

4.**Comment:** Line 269: “the stress‑strain curves for all fiber content levels exhibit a strain‑hardening type” specify “at −5°C” (already mentioned, but rephrasing could improve clarity).

**Response:** Thank you for your valuable advice. We revised lines 268 – 270 in the manuscript to explicitly state that strain hardening behavior was observed at a freezing temperature of-5 °C. The revised sentence reads: “At a freezing temperature of -5°C, the stress-strain curves of specimens with different fiber contents all exhibit a strain-hardening type.”

**a) The revised contents: At a freezing temperature of -5°C, the stress-strain curves of specimens with different fiber contents all exhibit a strain-hardening type.**

**b) The origin contents: At -5°C, the stress-strain curves for all fiber content levels exhibit a strain-hardening type**

5.**Comment:** Line 284: “energy dissipation through fiber‑frozen soil interface debonding” consider “debonding at the fiber‑frozen soil interface”.

**Response:** Thank you for your helpful suggestion. We have revised the phrase “fiber-frozen soil interface debonding” to “debonding at the fiber-frozen soil interface” in Lines 284–286 of the revised manuscript to improve clarity and readability.

**a) The revised contents: During this process, additional energy is dissipated through debonding at the fiber-frozen soil interface, fiber stretching, and fiber pullout, thereby significantly increasing the fracture energy of the specimen.**

**b) The origin contents: This process is accompanied by energy dissipation through fiber-frozen soil interface debonding, fiber stretching deformation, and fiber pullout, which significantly enhances the fracture energy of the specimen.**

6.**Comment:** Several sentences are overly long (e.g., lines 278‑286); breaking them would improve readability.

**Response:** Thank you for your helpful suggestion. We have revised the overly long sentences in Lines 280–289 to improve readability. In particular, the original long sentence describing the fiber-bridging mechanism has been divided into several shorter sentences. The revised text now more clearly describes microcrack initiation, fiber bridging across crack surfaces, tensile stress transfer, and crack-opening restriction.

**a) The revised contents: Beyond this threshold, fiber dispersion deteriorates markedly, resulting in fiber agglomeration. During tensile deformation of the fiber-reinforced frozen loess, microcracks initiate under loading. The fiber network then bridges the crack surfaces and bears part of the tensile stress. This bridging action restricts crack opening and delays crack propagation. During this process, additional energy is dissipated through debonding at the fiber-frozen soil interface, fiber stretching, and fiber pullout, thereby significantly increasing the fracture energy of the specimen. Meanwhile, the presence of fibers improves the stress transfer pathways within the soil matrix. Consequently, local stress concentration is alleviated, and the initiation and propagation of the dominant crack are delayed.**

**b) The origin contents: Beyond this threshold, the dispersion of fibers deteriorates sharply, resulting in the agglomeration of fibers. In the process of stress deformation of improved frozen loess, the fiber network crosses the crack surface through an effective fiber bridging effect when the sample is loaded to produce microcracks, withstands tensile stress, and limits crack opening. This process is accompanied by energy dissipation through fiber-frozen soil interface debonding, fiber stretching deformation, and fiber pullout, which significantly enhances the fracture energy of the specimen. Simultaneously, the presence of fibers optimizes stress transfer pathways, facilitating localized stress redistribution and delaying the initiation and propagation of the primary controlling crack.**

7.**Comment:** The manuscript uses both “fiber” and “fibre” (e.g., Figure 15 caption “Fibre Parameters”) please standardise to “fiber” (US spelling) or “fibre” consistently. PLOS ONE does not mandate a specific variant, but internal consistency is needed.

**Response:** Thank you for your helpful comment. We revised the manuscript to adopt the spelling "fiber."

8.**Comment:** Figures 12 and 13 are mentioned but not visible in the PDF. Ensure that high‑resolution versions with all annotations (error bars, regression lines, equations, R²) are uploaded.

**Response:** Thank you for your careful comment. We apologize for the display problem of Figures 12 and 13 in the previous PDF version. We have carefully checked the final compiled PDF and re-uploaded high-resolution versions of Figures 12 and 13. The updated figures now display correctly and include the required annotations, such as error bars and regression lines. In addition, the corresponding regression equations and R^2^ values have been explicitly added to the captions of Figures 12 and 13 to ensure quantitative reproducibility. We have also confirmed that all annotations are clearly visible in the revised PDF.

9.**Comment:** Figure 11 now includes significance letters (a‑d) – good. However, the caption should explain that different letters indicate statistically significant differences (p < 0.05) based on Tukey’s HSD.

**Response:** Thank you for your helpful comment. We have revised the caption of Fig. 11 to explain the meaning of the significance letters. The revised caption states that different lowercase letters above the bars indicate statistically significant differences among groups according to Tukey’s HSD test (p < 0.05). This revision has been made in the revised manuscript, Lines 386–388.

**The revised contents: Fig. 11. Effect of polypropylene fibre content on the tensile strength of frozen loess at -5°C and a loading rate of 1.57 mm/min. Different lowercase letters above the bars indicate statistically significant differences among groups according to Tukey’s HSD test (p < 0.05).**

**The origin contents: Fig. 11 Effect of Polypropylene Fiber Parameters on the Tensile Strength of Frozen Loess at -5°C and 1.57 mm/min.**

10.Table 1: The formatting of temperature ranges (e.g., “-2, -3, -5”) is inconsistent; consider using “−2, −3, −5” with proper minus signs.

**Response:** Thank you for your helpful comment. We have carefully checked the manuscript and revised the formatting of negative temperature values throughout the text, figures, tables, and captions. The hyphen-minus symbol “-” has been replaced with the proper minus sign “-” where negative temperatures are reported, such as “-2, -3, and -5 °C”. This ensures consistent and correct formatting throughout the revised manuscript.

11.Page 39-40: The text for Figure 15 appears garbled (repeated numbers). Please check the final compiled PDF.

**Response:** Thank you for your careful comment. We apologize for the formatting problem in the previous compiled PDF. We have carefully checked Pages 39–40 and corrected the garbled text associated with Figure 15. The repeated numbers have been removed, and Figure 15, including its caption and annotations, now displays correctly in the final compiled PDF. We have also rechecked the surrounding text and figure layout to ensure that no similar formatting errors remain.

**The revised contents:**

**Fig. 15 Effect of Fiber Parameters on Pore Number and Proportion of Pore Area.**

**The origin contents:**

**Fig. 15 Effect of Fiber Parameters on Pore Number and Proportion of Pore Area.**

12.Reference 9: “Unknown” as journal – please update if possible, or keep as is but note that it is an old report.

**Response:** Thank you for your helpful comment. We carefully checked Reference 9 and confirmed that it is not a journal article but an old technical report published by the U.S. Army Cold Regions Research and Engineering Laboratory. Therefore, we have updated the reference by replacing “Unknown” with the report information. In the revised manuscript, Reference 9 is now cited as an old technical report, namely CRREL Report RR350.

**The revised contents: Haynes FD, Karalius JA, Kalafut J. Strain rate effect on the strength of frozen silt. Hanover, NH: U.S. Army Cold Regions Research and Engineering Laboratory; 1975. CRREL Report RR 350. Available from:** [**https://ui.adsabs.harvard.edu/abs/1975sesf.rept.....H**](https://ui.adsabs.harvard.edu/abs/1975sesf.rept.....H)

**The origin contents: Haynes FD, Karalius JA, Kalafut J. Strain effect on the strength of frozen silt. Unknown. 1975 Dec. Available: https://ui.adsabs.harvard.edu/abs/1975sesf.rept.....H**
