# Supplementary material for: Study on tensile mechanical response and microstructure of polypropylene fiber reinforced loess under freezing
Source: PLoS One. 2026 Jun 9;21(6):e0350932. doi: 10.1371/journal.pone.0350932 (PMC13249173; doi:10.1371/journal.pone.0350932)
Supplement: Peer review history

# Peer review: Decision Letter 0

**DOI:** 10.1371/journal.pone.0350932.r001

**Authors:** Shamshad Alam

23 Feb 2026

PONE-D-26-00128Study on tensile mechanical response and microstructure of polypropylene fiber reinforced loess under freezingPLOS One

Dear Dr. He,

Thank you for submitting your manuscript to PLOS ONE. After careful consideration, we feel that it has merit but does not fully meet PLOS ONE’s publication criteria as it currently stands. Therefore, we invite you to submit a revised version of the manuscript that addresses the points raised during the review process.

Please submit your revised manuscript by Apr 09 2026 11:59PM. If you will need more time than this to complete your revisions, please reply to this message or contact the journal office at plosone@plos.org. When you're ready to submit your revision, log on to https://www.editorialmanager.com/pone/ and select the 'Submissions Needing Revision' folder to locate your manuscript file.

Please include the following items when submitting your revised manuscript:

- A letter that responds to each point raised by the academic editor and reviewer(s). You should upload this letter as a separate file labeled 'Response to Reviewers'.
- A marked-up copy of your manuscript that highlights changes made to the original version. You should upload this as a separate file labeled 'Revised Manuscript with Track Changes'.
- An unmarked version of your revised paper without tracked changes. You should upload this as a separate file labeled 'Manuscript'.

If you would like to make changes to your financial disclosure, please include your updated statement in your cover letter. Guidelines for resubmitting your figure files are available below the reviewer comments at the end of this letter.

If applicable, we recommend that you deposit your laboratory protocols in protocols.io to enhance the reproducibility of your results. Protocols.io assigns your protocol its own identifier (DOI) so that it can be cited independently in the future. For instructions see: https://journals.plos.org/plosone/s/submission-guidelines#loc-laboratory-protocols. Additionally, PLOS ONE offers an option for publishing peer-reviewed Lab Protocol articles, which describe protocols hosted on protocols.io. Read more information on sharing protocols at https://plos.org/protocols?utm_medium=editorial-email&utm_source=authorletters&utm_campaign=protocols.

We look forward to receiving your revised manuscript.

Kind regards,

Shamshad Alam, PhD

Academic Editor

PLOS One

Journal Requirements:

When submitting your revision, we need you to address these additional requirements.

1. Please ensure that your manuscript meets PLOS ONE's style requirements, including those for file naming. The PLOS ONE style templates can be found at

https://journals.plos.org/plosone/s/file?id=wjVg/PLOSOne_formatting_sample_main_body.pdf and

https://journals.plos.org/plosone/s/file?id=ba62/PLOSOne_formatting_sample_title_authors_affiliations.pdf

2. In your Methods section, please provide additional information regarding the permits you obtained for the work. Please ensure you have included the full name of the authority that approved the field site access and, if no permits were required, a brief statement explaining why.

3. Please note that PLOS One has specific guidelines on code sharing for submissions in which author-generated code underpins the findings in the manuscript. In these cases, we expect all author-generated code to be made available without restrictions upon publication of the work. Please review our guidelines at https://journals.plos.org/plosone/s/materials-and-software-sharing#loc-sharing-code and ensure that your code is shared in a way that follows best practice and facilitates reproducibility and reuse.

4. We note that your Data Availability Statement is currently as follows: “All relevant data are within the manuscript and its Supporting Information files.”

Please confirm at this time whether or not your submission contains all raw data required to replicate the results of your study. Authors must share the “minimal data set” for their submission. PLOS defines the minimal data set to consist of the data required to replicate all study findings reported in the article, as well as related metadata and methods (https://journals.plos.org/plosone/s/data-availability#loc-minimal-data-set-definition).

For example, authors should submit the following data:

- The values behind the means, standard deviations and other measures reported;

- The values used to build graphs;

- The points extracted from images for analysis.

Authors do not need to submit their entire data set if only a portion of the data was used in the reported study.

If your submission does not contain these data, please either upload them as Supporting Information files or deposit them to a stable, public repository and provide us with the relevant URLs, DOIs, or accession numbers. For a list of recommended repositories, please see https://journals.plos.org/plosone/s/recommended-repositories.

If there are ethical or legal restrictions on sharing a de-identified data set, please explain them in detail (e.g., data contain potentially sensitive information, data are owned by a third-party organization, etc.) and who has imposed them (e.g., an ethics committee). Please also provide contact information for a data access committee, ethics committee, or other institutional body to which data requests may be sent. If data are owned by a third party, please indicate how others may request data access.

5. Please upload a copy of Supporting Information which you refer to in your text on page 27-28

6. We note that Figures 1, 3, 4 and 12 in your submission contain copyrighted images. All PLOS content is published under the Creative Commons Attribution License (CC BY 4.0), which means that the manuscript, images, and Supporting Information files will be freely available online, and any third party is permitted to access, download, copy, distribute, and use these materials in any way, even commercially, with proper attribution. For more information, see our copyright guidelines: http://journals.plos.org/plosone/s/licenses-and-copyright.

We require you to either (1) present written permission from the copyright holder to publish these figures specifically under the CC BY 4.0 license, or (2) remove the figures from your submission:

(1) You may seek permission from the original copyright holder of Figures 1, 3, 4 and 12 to publish the content specifically under the CC BY 4.0 license.

We recommend that you contact the original copyright holder with the Content Permission Form (http://journals.plos.org/plosone/s/file?id=7c09/content-permission-form.pdf) and the following text:

“I request permission for the open-access journal PLOS ONE to publish XXX under the Creative Commons Attribution License (CCAL) CC BY 4.0 (http://creativecommons.org/licenses/by/4.0/). Please be aware that this license allows unrestricted use and distribution, even commercially, by third parties. Please reply and provide explicit written permission to publish XXX under a CC BY license and complete the attached form.”

Please upload the completed Content Permission Form or other proof of granted permissions as an "Other" file with your submission.

In the figure caption of the copyrighted figure, please include the following text: “Reprinted from [ref] under a CC BY license, with permission from [name of publisher], original copyright [original copyright year].”

(2) If you are unable to obtain permission from the original copyright holder to publish these figures under the CC BY 4.0 license or if the copyright holder’s requirements are incompatible with the CC BY 4.0 license, please either i) remove the figure or ii) supply a replacement figure that complies with the CC BY 4.0 license. Please check copyright information on all replacement figures and update the figure caption with source information. If applicable, please specify in the figure caption text when a figure is similar but not identical to the original image and is therefore for illustrative purposes only

7. If the reviewer comments include a recommendation to cite specific previously published works, please review and evaluate these publications to determine whether they are relevant and should be cited. There is no requirement to cite these works unless the editor has indicated otherwise.

[Note: HTML markup is below. Please do not edit.]

Reviewers' comments:

Reviewer's Responses to Questions

**Comments to the Author**

1. Is the manuscript technically sound, and do the data support the conclusions?

The manuscript must describe a technically sound piece of scientific research with data that supports the conclusions. Experiments must have been conducted rigorously, with appropriate controls, replication, and sample sizes. The conclusions must be drawn appropriately based on the data presented.

Reviewer #1: Yes

Reviewer #2: Partly

**********

2. Has the statistical analysis been performed appropriately and rigorously?

Reviewer #1: Yes

Reviewer #2: No

**********

3. Have the authors made all data underlying the findings in their manuscript fully available?

The PLOS Data policy requires authors to make all data underlying the findings described in their manuscript fully available without restriction, with rare exception (please refer to the Data Availability Statement in the manuscript PDF file). The data should be provided as part of the manuscript or its supporting information, or deposited to a public repository. For example, in addition to summary statistics, the data points behind means, medians and variance measures should be available. If there are restrictions on publicly sharing data—e.g. participant privacy or use of data from a third party—those must be specified.

Reviewer #1: Yes

Reviewer #2: No

**********

4. Is the manuscript presented in an intelligible fashion and written in standard English?

PLOS ONE does not copyedit accepted manuscripts, so the language in submitted articles must be clear, correct, and unambiguous. Any typographical or grammatical errors should be corrected at revision, so please note any specific errors here.

Reviewer #1: Yes

Reviewer #2: No

**********

5. Review Comments to the Author

Please use the space provided to explain your answers to the questions above. You may also include additional comments for the author, including concerns about dual publication, research ethics, or publication ethics. (Please upload your review as an attachment if it exceeds 20,000 characters)

Reviewer #1: 1. In line 241, is the failure modes of test specimens showing the same brittle fracture failure at different moisture content?

2. Please providing further information about the test specimen, such as dimensions, shapes or etc

3. In fig 6, the stress-deformation curve of the soil sample under-5℃ does not work as a kind of brittle material, this may be contradictory to the conclusion in line 241.

4. There is also the same doubt in Fig 7

5. How to get an appropriate amount of fiber fully motivating the role in filling small pores and fiber reinforcing densification?

Reviewer #2: Review Comments to the Author

The manuscript investigates the tensile behavior and microstructural characteristics of polypropylene fiber-reinforced loess under freezing conditions. The topic is relevant for cold-region geotechnical engineering, and the experimental effort is appreciated. The study has potential practical value. However, several issues related to experimental rigor, data presentation, and clarity should be addressed to improve the scientific quality of the paper.

Major Comments

1. The manuscript does not clearly indicate the number of specimens tested for each condition. Please report the number of replicates and include measures of variability such as standard deviation or error bars. Without this information, it is difficult to assess the reliability of the reported results.

2. The conclusion that 0.3% fiber content is optimal should be supported by statistical analysis. Please provide appropriate statistical tests (e.g., ANOVA or similar) to demonstrate that the differences between fiber contents are significant.

3. The novelty of the study should be clarified. Similar trends regarding fiber dosage effects, temperature dependence, and loading rate influence have been reported in previous studies. The authors should clearly state what new contribution this work provides.

4. The self-developed tensile testing device is an important part of the study, but its accuracy and reliability are not discussed. Please provide information on calibration, validation, or comparison with existing methods.

The experimental procedure requires more detail, particularly regarding specimen preparation, fiber mixing and dispersion, freezing conditions (freezing duration and temperature control), and measures taken to ensure uniformity.

5. The statements that tensile strength varies exponentially with temperature and linearly with loading rate should be supported by regression equations and goodness-of-fit values.

6. The SEM analysis is mainly descriptive. If possible, please include quantitative information or provide a clearer explanation of how the observed microstructure supports the mechanical results.

7. Raw experimental data (e.g., stress–strain curves or numerical values) should be provided as Supporting Information in accordance with data availability requirements.

Minor Comments

(i) The manuscript would benefit from professional English language editing to improve grammar and readability.

(ii) Some parts of the Results and Discussion repeat similar descriptions and should be shortened for clarity.

(iii) Please use consistent terminology throughout the manuscript (e.g., stress–strain vs. stress–deformation).

(iv) The Conclusions section should be revised to avoid overly strong statements and to reflect the limitations of the experimental study.

Overall comment

The study addresses a relevant engineering problem and shows promise, but improvements in statistical reporting, experimental description, data transparency, and presentation are necessary before the manuscript can be considered for publication.

**********

6. PLOS authors have the option to publish the peer review history of their article (what does this mean?). If published, this will include your full peer review and any attached files.

If you choose “no”, your identity will remain anonymous but your review may still be made public.

**Do you want your identity to be public for this peer review?** For information about this choice, including consent withdrawal, please see our Privacy Policy.

Reviewer #1: **Yes:** JUN LIN

Reviewer #2: No

**********

[NOTE: If reviewer comments were submitted as an attachment file, they will be attached to this email and accessible via the submission site. Please log into your account, locate the manuscript record, and check for the action link "View Attachments". If this link does not appear, there are no attachment files.]

To ensure your figures meet our technical requirements, please review our figure guidelines: https://journals.plos.org/plosone/s/figures

You may also use PLOS’s free figure tool, NAAS, to help you prepare publication quality figures: https://journals.plos.org/plosone/s/figures#loc-tools-for-figure-preparation.

NAAS will assess whether your figures meet our technical requirements by comparing each figure against our figure specifications.

# Author comment: Author response to Decision Letter 1

**DOI:** 10.1371/journal.pone.0350932.r002

21 Mar 2026

Academic editor's requirements:

1.Please ensure that your manuscript meets PLOS ONE's style requirements, including those for file naming.

Response: Based on the downloaded template files, we have revised the article to ensure meet PLOS ONE's style requirements.

2.In your Methods section, please provide additional information regarding the permits you obtained for the work.Please ensure you have included the full name of the authority that approved the field site access and, if no permits were required, a brief statement explaining why.

Response: Thank you for your comment. We would like to clarify that no permit was required for this study.

The soil used in this study was collected from Daqingshan in Anning District, Lanzhou, China. This site is a non-protected public area and does not involve any endangered or protected species. Therefore, no specific fieldwork or collection permits were required for this study. Regarding the details of the soil sampling area, we have made relevant revisions in Section 2.2 ‘Test Soils and Sample Preparation’ of the paper; please refer to lines 160–162.

3.Please note that PLOS One has specific guidelines on code sharing for submissions in which author-generated code underpins the findings in the manuscript. In these cases, we expect all author-generated code to be made available without restrictions upon publication of the work.

Response: Thank you for the reminder regarding PLOS One’s code sharing policy. We would like to clarify that this study did not involve any author-generated code or specialized software scripts to underpin the findings. All data analysis and statistical tests (e.g., ANOVA, plotting in Origin) were performed using standard, commercially available software. Therefore, the code-sharing requirement is not applicable to this manuscript.

4.We note that your Data Availability Statement is currently as follows: “All relevant data are within the manuscript and its Supporting Information files.”Please confirm at this time whether or not your submission contains all raw data required to replicate the results of your study. Authors must share the “minimal data set” for their submission. PLOS defines the minimal data set to consist of the data required to replicate all study findings reported in the article, as well as related metadata and methods

Response: We apologize that we did not specify where to find the minimal dataset on which our experimental results are based. We have uploaded the data into an online database, which can be found at the link below.

https://doi.org/10.6084/m9.figshare.31812757

5.Please upload a copy of Supporting Information which you refer to in your text on page 27-28

Response: Thank you for pointing this out. We apologize for the oversight in the initial submission. We have now uploaded the Supporting Information file to the submission system. This file contains all the additional data and figures referenced on pages 27-28 of the manuscript.

6.We note that Figures 1, 3, 4 and 12 in your submission contain copyrighted images. All PLOS content is published under the Creative Commons Attribution License (CC BY 4.0), which means that the manuscript, images, and Supporting Information files will be freely available online, and any third party is permitted to access, download, copy, distribute, and use these materials in any way, even commercially, with proper attribution. For more information, see our copyright guidelines: http://journals.plos.org/plosone/s/licenses-and-copyright.

Response: Thank you for the reminder regarding copyright. We would like to clarify that Figures 1, 3, 4, and 12 are original works created by the authors of this study.

7.If the reviewer comments include a recommendation to cite specific previously published works, please review and evaluate these publications to determine whether they are relevant and should be cited. There is no requirement to cite these works unless the editor has indicated otherwise.

Response: We have noted the policy regarding the citation of previously published works. We confirm that all citations in the revised manuscript have been evaluated for their relevance and necessity to support our findings.

Reviewer #1:

1.Comment: In line 241, is the failure modes of test specimens showing the same brittle fracture failure at different moisture content?

Response: We sincerely thank the reviewer for this insightful comment. First, we would like to emphasize that in our experimental design, the moisture content of all samples was strictly controlled as a constant variable at 16.2% (see Table 1 for details). Therefore, the change in failure mode is not driven by moisture content. However, we fully agree with your core criticism that describing all failure modes as “brittle fracture” is inaccurate. In the revised manuscript, we have accurately described how the fracture mode transitions from quasi-brittle to ductile behavior upon fiber incorporation, as detailed in lines 348–350.

2.Comment: Please providing further information about the test specimen, such as dimensions, shapes or etc

Response: We greatly appreciate your careful review and valuable feedback. We fully agree that the specific geometric details of the test specimens are crucial for understanding stress distribution and ensuring the reproducibility of the experiment. We sincerely apologize for the omission of these details in the original manuscript. We have now explicitly included these details regarding the shape and dimensions of the specimens in the revised manuscript, as described in lines 120–158.

3.Comment: In fig 6, the stress-deformation curve of the soil sample under-5℃ does not work as a kind of brittle material, this may be contradictory to the conclusion in line 241.

Response: We greatly appreciate your careful review and valuable suggestions. The stress-strain curves below -5°C (see Figure 6) clearly exhibit sustained deformation and strain hardening/softening characteristics, indicating that the material exhibits a ductile response rather than purely brittle behavior. The original vague statement on line 241 was indeed a general error that contradicted our data. We have corrected this in the revised manuscript (lines 348–350), highlighting the ductile transition to ensure strict consistency between the textual description and the experimental curves.

4.Comment: There is also the same doubt in Fig 7

Response: We thank the reviewers for their keen observations. Similar to the case in Figure 6, the stress-strain curves in Figure 7 under different loading rates also exhibit varying degrees of ductility and post-peak deformation. It is inaccurate to collectively refer to this phenomenon as “brittle fracture.” The original, vague statement in line 241 was indeed a error that contradicted our data. We have corrected this in the revised manuscript (lines 348–350), highlighting the ductility transition to ensure strict consistency between the textual description and the experimental curves.

5.Comment: How to get an appropriate amount of fiber fully motivating the role in filling small pores and fiber reinforcing densification?

Response: We sincerely thank the reviewers for raising these insightful and critical questions regarding the reinforcement mechanism. In our study, the “optimal amount” was determined by identifying a microstructural equilibrium point—specifically, the critical threshold between the formation of an effective spatial network and the onset of fiber aggregation. As discussed in Section 3.4 regarding the analysis of the reinforcement mechanism for polypropylene fibers: when the fiber content is below the optimal level, the fibers are too sparse to form an interconnected network, resulting in limited pore-filling effects. When the fiber content reaches the appropriate level (0.3%), the fibers are uniformly distributed and cross-overlap, effectively partitioning large pores and filling micropores. At this specific threshold, “fiber-reinforced compaction” is fully activated. Once the fiber content becomes too high (e.g., 0.4% or 0.5%), the limited spatial distance causes the fibers to become entangled and agglomerate. This agglomeration effect prevents soil particles from integrating into the network and creates large voids when the fibers are pulled out, ultimately weakening the structure. We have revised lines 448–452 to fully incorporate an explanation of this quantitative mechanism into the revised draft.

Reviewer #2:

1.Comment: The manuscript does not clearly indicate the number of specimens tested for each condition. Please report the number of replicates and include measures of variability such as standard deviation or error bars. Without this information, it is difficult to assess the reliability of the reported results.

Response: We greatly appreciate the reviewers’ rigorous comments regarding the reliability of our experiments. We sincerely apologize for failing to clearly explain the experimental replication process in the original manuscript.

During the experimental phase, we strictly adhered to the requirements for replication, conducting three parallel experiments (N=3) for each of the experimental conditions listed in Table 1 to ensure the reliability of the data. Furthermore, we performed a comprehensive statistical analysis of the replicate groups; the deviation between the single-test strength values and the average strength values from the three replicate tests did not exceed 8% under any experimental condition. The relative error between replicate samples was minimal, and the presented mean values accurately reflect the true trend of the parameters. To maintain the visual clarity of the complex stress–strain curves and avoid data redundancy, we have chosen to present the mean values in the figures.

Translated with DeepL.com (free version)To directly address your concerns and enhance the rigor of the manuscript, we have explicitly outlined the experimental replication procedure in lines 195–199.

2. Comment: The conclusion that 0.3% fiber content is optimal should be supported by statistical analysis. Please provide appropriate statistical tests (e.g., ANOVA or similar) to demonstrate that the differences between fiber contents are significant.

Response: We sincerely thank the reviewers for their rigorous and constructive suggestions. We fully agree that statistical analysis is crucial for validating our conclusions regarding the optimal fiber content.

Following your recommendation, we conducted a one-way analysis of variance (ANOVA) and Tukey’s post-hoc test to comprehensively assess the significance of differences in peak stress among the composite groups. The statistical results (F(4,10) = 68.71) indicate that fiber content has a significant effect on peak stress. Specifically, Tukey’s post-hoc analysis confirmed that the peak stress in the 0.3% group was significantly higher than in all other experimental groups (P < 0.05 for all pairwise comparisons with the 0.3% group). This provides strong statistical evidence that 0.3% is indeed the optimal fiber content.

We have updated the manuscript on lines 352–384 to incorporate these statistical findings. We have also revised Figure 11, adding standard error bars and statistical significance labels (a–d) to visually convey these results.

3. Comment: The novelty of the study should be clarified. Similar trends regarding fiber dosage effects, temperature dependence, and loading rate influence have been reported in previous studies. The authors should clearly state what new contribution this work provides.

Response: We sincerely thank this reviewer for their critical and thought-provoking comments. We agree that the macroscopic trends observed in our study—such as the increase in strength at the optimal fiber dosage and the temperature-dependent strengthening effect—are highly consistent with the existing literature. In fact, this consistency further validates the reliability and accuracy of our experimental testing system. However, we respectfully emphasize that the primary novelty and core contribution of this work extend far beyond these macroscopic trends.

① It focuses on the tensile strength of fiber-reinforced frozen loess—a specific material system relevant to engineering in cold regions that has previously received insufficient attention.

② A custom-designed direct tensile testing apparatus was employed, thereby overcoming the limitations of the indirect testing methods commonly used in previous studies.

③ This study systematically examined the interactions among fiber content, freezing temperature, and loading rate, revealing how these factors influence one another and, in turn, affect mechanical properties.

④ The study provides microstructural evidence explaining the transition from brittle to ductile fracture and the “optimal fiber content” phenomenon (i.e., 0.3% fiber content)—at this level, appropriate fiber incorporation fills voids and forms a reinforcing network, whereas excessive fiber leads to agglomeration and weakening.

To better highlight the innovation and contributions of this study, we have revised the Introduction (lines 100–109).

4. Comment: The self-developed tensile testing device is an important part of the study, but its accuracy and reliability are not discussed. Please provide information on calibration, validation, or comparison with existing methods.

Response: We sincerely thank this reviewer for their rigorous and highly professional comments. We fully agree that validating the in-house developed equipment is a key prerequisite for ensuring the credibility of the entire study. We conducted validation of the experimental data immediately after the tensile tester was completed. To directly address your concerns and clearly demonstrate the reliability of the equipment, we have now included details of the equipment validation in the revised manuscript. Specifically, we would like to clarify the following:

① Calibration: All core measurement components (such as high-precision load cells and displacement sensors) undergo rigorous factory calibration and are zeroed before each test to ensure sensor accuracy.

② Comparison with Existing Methods: Most importantly, during the preliminary research phase, we conducted systematic comparative validation using the classic split-tensile test as a benchmark. The direct tensile strength data obtained from our in-house developed equipment yielded a ratio of 0.86 compared to the results from the classic split tensile test, which is consistent with existing research findings (Perras et al. found that the strength from direct tensile tests is typically only 70%–90% of that from the classic Brazilian split test, doi:10.1007/s10706-014-9732-0). This cross-validation effectively demonstrates the mechanical accuracy of our equipment.

We have added a dedicated section in the experimental design section (lines 201–227) to provide a detailed explanation of this comparative validation and the reliability of the equipment, in order to maintain transparency for the reader.

The experimental procedure requires more detail, particularly regarding specimen preparation, fiber mixing and dispersion, freezing conditions (freezing duration and temperature control), and measures taken to ensure uniformity.

Response: We sincerely thank the reviewers for pointing out this important omission. We agree that a detailed description of the experimental procedures is essential for the reproducibility and reliability of the study. We have added further details to the section “2.2 Soil Samples and Sample Preparation.” Please refer to lines 179–187 for the relevant information.

5. Comment: The statements that tensile strength varies exponentially with temperature and linearly with loading rate should be supported by regression equations and goodness-of-fit values.

Response: We are very grateful to the reviewers for their constructive and thorough feedback. We fully agree that providing precise mathematical models and their goodness-of-fit statistics significantly enhances the quantitative credibility of our conclusions. Following your suggestion, we have added the linear regression equations and included the goodness-of-fit values in Figures 12 and 13 on lines 385–389 of the revised manu

Attachment Submitted filename: Response to reviewers.docx

# Peer review: Decision Letter 1

**DOI:** 10.1371/journal.pone.0350932.r003

**Authors:** Shamshad Alam

4 May 2026

<div>PONE-D-26-00128R1Study on tensile mechanical response and microstructure of polypropylene fiber reinforced loess under freezingPLOS One

Dear Dr. He,

Thank you for submitting your manuscript to PLOS ONE. After careful consideration, we feel that it has merit but does not fully meet PLOS ONE’s publication criteria as it currently stands. Therefore, we invite you to submit a revised version of the manuscript that addresses the points raised during the review process.

Please submit your revised manuscript by Jun 18 2026 11:59PM. If you will need more time than this to complete your revisions, please reply to this message or contact the journal office at plosone@plos.org. When you're ready to submit your revision, log on to https://www.editorialmanager.com/pone/ and select the 'Submissions Needing Revision' folder to locate your manuscript file.

Please include the following items when submitting your revised manuscript:

- A letter that responds to each point raised by the academic editor and reviewer(s). You should upload this letter as a separate file labeled 'Response to Reviewers'.
- A marked-up copy of your manuscript that highlights changes made to the original version. You should upload this as a separate file labeled 'Revised Manuscript with Track Changes'.
- An unmarked version of your revised paper without tracked changes. You should upload this as a separate file labeled 'Manuscript'.

If you would like to make changes to your financial disclosure, please include your updated statement in your cover letter. Guidelines for resubmitting your figure files are available below the reviewer comments at the end of this letter.

If applicable, we recommend that you deposit your laboratory protocols in protocols.io to enhance the reproducibility of your results. Protocols.io assigns your protocol its own identifier (DOI) so that it can be cited independently in the future. For instructions see: https://journals.plos.org/plosone/s/submission-guidelines#loc-laboratory-protocols. Additionally, PLOS ONE offers an option for publishing peer-reviewed Lab Protocol articles, which describe protocols hosted on protocols.io. Read more information on sharing protocols at https://plos.org/protocols?utm_medium=editorial-email&utm_source=authorletters&utm_campaign=protocols.

As the corresponding author, your ORCID iD is verified in the submission system and will appear in the published article. PLOS supports the use of ORCID, and we encourage all coauthors to register for an ORCID iD and use it as well. Please encourage your coauthors to verify their ORCID iD within the submission system before final acceptance, as unverified ORCID iDs will not appear in the published article. *Only* the individual author can complete the verification step; PLOS staff *cannot* verify ORCID iDs on behalf of authors.

We look forward to receiving your revised manuscript.

Kind regards,

Shamshad Alam, PhD

Academic Editor

PLOS One

Journal Requirements:

1. If the reviewer comments include a recommendation to cite specific previously published works, please review and evaluate these publications to determine whether they are relevant and should be cited. There is no requirement to cite these works unless the editor has indicated otherwise.

2. Please review your reference list to ensure that it is complete and correct. If you have cited papers that have been retracted, please include the rationale for doing so in the manuscript text, or remove these references and replace them with relevant current references. Any changes to the reference list should be mentioned in the rebuttal letter that accompanies your revised manuscript. If you need to cite a retracted article, indicate the article’s retracted status in the References list and also include a citation and full reference for the retraction notice.

[Note: HTML markup is below. Please do not edit.]

Reviewers' comments:

Reviewer's Responses to Questions

**Comments to the Author**

1. If the authors have adequately addressed your comments raised in a previous round of review and you feel that this manuscript is now acceptable for publication, you may indicate that here to bypass the “Comments to the Author” section, enter your conflict of interest statement in the “Confidential to Editor” section, and submit your "Accept" recommendation.

Reviewer #1: All comments have been addressed

Reviewer #2: (No Response)

**********

2. Is the manuscript technically sound, and do the data support the conclusions?

The manuscript must describe a technically sound piece of scientific research with data that supports the conclusions. Experiments must have been conducted rigorously, with appropriate controls, replication, and sample sizes. The conclusions must be drawn appropriately based on the data presented.

Reviewer #1: Yes

Reviewer #2: Partly

**********

3. Has the statistical analysis been performed appropriately and rigorously?

Reviewer #1: Yes

Reviewer #2: Yes

**********

4. Have the authors made all data underlying the findings in their manuscript fully available?

The PLOS Data policy requires authors to make all data underlying the findings described in their manuscript fully available without restriction, with rare exception (please refer to the Data Availability Statement in the manuscript PDF file). The data should be provided as part of the manuscript or its supporting information, or deposited to a public repository. For example, in addition to summary statistics, the data points behind means, medians and variance measures should be available. If there are restrictions on publicly sharing data—e.g. participant privacy or use of data from a third party—those must be specified.

Reviewer #1: Yes

Reviewer #2: No

**********

5. Is the manuscript presented in an intelligible fashion and written in standard English?

PLOS ONE does not copyedit accepted manuscripts, so the language in submitted articles must be clear, correct, and unambiguous. Any typographical or grammatical errors should be corrected at revision, so please note any specific errors here.

Reviewer #1: Yes

Reviewer #2: No

**********

6. Review Comments to the Author

Please use the space provided to explain your answers to the questions above. You may also include additional comments for the author, including concerns about dual publication, research ethics, or publication ethics. (Please upload your review as an attachment if it exceeds 20,000 characters)

Reviewer #1: All comments are accepted and the manuscript has been rivesed well. It is advised to published in this journal.

Reviewer #2: In Section 3.1 Please revise line 232‑233 to clearly distinguish the brittle failure of plain frozen loess from the ductile, multiple‑cracking failure of fiber‑reinforced specimens.

Please replace the current Data Availability Statement with the Figshare DOI and confirm that the data are publicly accessible.

Please explicitly write the exponential regression equation (with constants and R²) for Figure 12, and the linear equation (slope, intercept, R²) for Figure 13 in the respective figure captions or in the Results section (lines 385‑389). This is essential for quantitative reproducibility.

Line 269: “the stress‑strain curves for all fiber content levels exhibit a strain‑hardening type” specify “at −5°C” (already mentioned, but rephrasing could improve clarity).

Line 284: “energy dissipation through fiber‑frozen soil interface debonding” consider “debonding at the fiber‑frozen soil interface”.

Several sentences are overly long (e.g., lines 278‑286); breaking them would improve readability.

The manuscript uses both “fiber” and “fibre” (e.g., Figure 15 caption “Fibre Parameters”) please standardise to “fiber” (US spelling) or “fibre” consistently. PLOS ONE does not mandate a specific variant, but internal consistency is needed.

Figures 12 and 13 are mentioned but not visible in the PDF. Ensure that high‑resolution versions with all annotations (error bars, regression lines, equations, R²) are uploaded.

Figure 11 now includes significance letters (a‑d) – good. However, the caption should explain that different letters indicate statistically significant differences (p < 0.05) based on Tukey’s HSD.

Table 1: The formatting of temperature ranges (e.g., “-2, -3, -5”) is inconsistent; consider using “−2, −3, −5” with proper minus signs.

Page 39‑40: The text for Figure 15 appears garbled (repeated numbers). Please check the final compiled PDF.

Reference 9: “Unknown” as journal – please update if possible, or keep as is but note that it is an old report.

**********

7. PLOS authors have the option to publish the peer review history of their article (what does this mean?). If published, this will include your full peer review and any attached files.

If you choose “no”, your identity will remain anonymous but your review may still be made public.

**Do you want your identity to be public for this peer review?** For information about this choice, including consent withdrawal, please see our Privacy Policy.

Reviewer #1: **Yes:** JUN LIN

Reviewer #2: No

**********

[NOTE: If reviewer comments were submitted as an attachment file, they will be attached to this email and accessible via the submission site. Please log into your account, locate the manuscript record, and check for the action link "View Attachments". If this link does not appear, there are no attachment files.]

To ensure your figures meet our technical requirements, please review our figure guidelines: https://journals.plos.org/plosone/s/figures

You may also use PLOS’s free figure tool, NAAS, to help you prepare publication quality figures: https://journals.plos.org/plosone/s/figures#loc-tools-for-figure-preparation.

NAAS will assess whether your figures meet our technical requirements by comparing each figure against our figure specifications.

# Author comment: Author response to Decision Letter 2

**DOI:** 10.1371/journal.pone.0350932.r004

14 May 2026

Reviewer #2:

1.Comment: In Section 3.1 Please revise line 232-233 to clearly distinguish the brittle failure of plain frozen loess from the ductile, multiple-cracking failure of fiber-reinforced specimens.

Response: Thank you for your valuable suggestion. We agree that the distinction between the failure modes of plain frozen loess and fiber-reinforced specimens should be stated more clearly. Accordingly, we have revised Section 3.1, Lines 230–232, to clarify that the plain frozen loess specimens exhibited typical brittle failure, characterized by abrupt tensile fracture and the formation of a dominant main crack. In contrast, the fiber-reinforced specimens showed a more ductile and progressive multiple-cracking failure pattern due to the crack-bridging and pull-out effects of polypropylene fibers. The corresponding discussion has been added in the revised.

a) The revised contents: Frozen loess exhibits typical brittle fracture characteristics under tensile action, while the failure pattern of frozen loess modified by polypropylene fiber gradually changes from single brittle fracture to multi-crack and gradual failure.

b) The origin contents: The tensile test failure of frozen loess and frozen polypropylene fiber-modified loess is brittle fracture failure.

2.Comment: Please replace the current Data Availability Statement with the Figshare DOI and confirm that the data are publicly accessible.

Response: Thank you for your helpful comment. We have revised the Data Availability Statement accordingly. The data supporting the findings of this study have been deposited in Figshare and are publicly accessible at https://doi.org/10.6084/m9.figshare.31812757. We have confirmed that the dataset is publicly accessible. This revision has been made in the revised manuscript, Lines 505–506.

a) The revised contents: The data supporting the findings of this study have been deposited in Figshare and are publicly accessible at https://doi.org/10.6084/m9.figshare.31812757.

b) The origin contents: Data will be made available on request.

3.Comment: Please explicitly write the exponential regression equation (with constants and R²) for Figure 12, and the linear equation (slope, intercept, R²) for Figure 13 in the respective figure captions or in the Results section (lines 385-389). This is essential for quantitative reproducibility.

Response: Thank you for your valuable input. We agree that explicitly proposing regression equations is important for quantitative reproducibility. In line 390-399, we have modified the annotation of Figure 12 and Figure 13 to explicitly write the regression equation (with constant terms and R²).

a) The revised contents: Fig. 12. Effect of freezing temperature on the tensile strength of fiber-reinforced frozen loess at a loading rate of 1.57 mm/min and a fiber content of 0.3%.The exponential regression equation is y=6.73+3.26×0.43x, with R2=0.99, where y is the tensile strength in kPa and x is the freezing temperature in ℃.

Fig. 13 Relationship curve between tensile strength and loading rate.The fitted linear equations were y=189.5+12.4x, R2=0.86 for the -5℃，0.3% fiber-reinforced loess; y=110.6+11.67x, R2=0.99, for frozen loess at -5 ℃; y=9.6+2.99x, R2=0.92, for frozen loess at -3 ℃; y=5.3+2.87x, R2=0.97, for frozen loess at -2 ℃; where y is the tensile strength in kPa and x is the loading rate in mm/min.

b) The origin contents: Fig. 12 Effect of modified loess freezing temperature at a loading rate of 1.57 mm/min and 0.3% fiber content on loess tensile strength.

Fig. 13 Relationship curve between tensile strength and loading rate.

4.Comment: Line 269: “the stress-strain curves for all fiber content levels exhibit a strain-hardening type” specify “at −5°C” (already mentioned, but rephrasing could improve clarity).

Response: Thank you for your valuable advice. We revised lines 268 – 270 in the manuscript to explicitly state that strain hardening behavior was observed at a freezing temperature of-5 °C. The revised sentence reads: “At a freezing temperature of -5°C, the stress-strain curves of specimens with different fiber contents all exhibit a strain-hardening type.”

a) The revised contents: At a freezing temperature of -5°C, the stress-strain curves of specimens with different fiber contents all exhibit a strain-hardening type.

b) The origin contents: At -5°C, the stress-strain curves for all fiber content levels exhibit a strain-hardening type

5.Comment: Line 284: “energy dissipation through fiber-frozen soil interface debonding” consider “debonding at the fiber-frozen soil interface”.

Response: Thank you for your helpful suggestion. We have revised the phrase “fiber-frozen soil interface debonding” to “debonding at the fiber-frozen soil interface” in Lines 284–286 of the revised manuscript to improve clarity and readability.

a) The revised contents: During this process, additional energy is dissipated through debonding at the fiber-frozen soil interface, fiber stretching, and fiber pullout, thereby significantly increasing the fracture energy of the specimen.

b) The origin contents: This process is accompanied by energy dissipation through fiber-frozen soil interface debonding, fiber stretching deformation, and fiber pullout, which significantly enhances the fracture energy of the specimen.

6.Comment: Several sentences are overly long (e.g., lines 278-286); breaking them would improve readability.

Response: Thank you for your helpful suggestion. We have revised the overly long sentences in Lines 280–289 to improve readability. In particular, the original long sentence describing the fiber-bridging mechanism has been divided into several shorter sentences. The revised text now more clearly describes microcrack initiation, fiber bridging across crack surfaces, tensile stress transfer, and crack-opening restriction.

a) The revised contents: Beyond this threshold, fiber dispersion deteriorates markedly, resulting in fiber agglomeration. During tensile deformation of the fiber-reinforced frozen loess, microcracks initiate under loading. The fiber network then bridges the crack surfaces and bears part of the tensile stress. This bridging action restricts crack opening and delays crack propagation. During this process, additional energy is dissipated through debonding at the fiber-frozen soil interface, fiber stretching, and fiber pullout, thereby significantly increasing the fracture energy of the specimen. Meanwhile, the presence of fibers improves the stress transfer pathways within the soil matrix. Consequently, local stress concentration is alleviated, and the initiation and propagation of the dominant crack are delayed.

b) The origin contents: Beyond this threshold, the dispersion of fibers deteriorates sharply, resulting in the agglomeration of fibers. In the process of stress deformation of improved frozen loess, the fiber network crosses the crack surface through an effective fiber bridging effect when the sample is loaded to produce microcracks, withstands tensile stress, and limits crack opening. This process is accompanied by energy dissipation through fiber-frozen soil interface debonding, fiber stretching deformation, and fiber pullout, which significantly enhances the fracture energy of the specimen. Simultaneously, the presence of fibers optimizes stress transfer pathways, facilitating localized stress redistribution and delaying the initiation and propagation of the primary controlling crack.

7.Comment: The manuscript uses both “fiber” and “fibre” (e.g., Figure 15 caption “Fibre Parameters”) please standardise to “fiber” (US spelling) or “fibre” consistently. PLOS ONE does not mandate a specific variant, but internal consistency is needed.

Response: Thank you for your helpful comment. We revised the manuscript to adopt the spelling "fiber."

8.Comment: Figures 12 and 13 are mentioned but not visible in the PDF. Ensure that high-resolution versions with all annotations (error bars, regression lines, equations, R²) are uploaded.

Response: Thank you for your careful comment. We apologize for the display problem of Figures 12 and 13 in the previous PDF version. We have carefully checked the final compiled PDF and re-uploaded high-resolution versions of Figures 12 and 13. The updated figures now display correctly and include the required annotations, such as error bars and regression lines. In addition, the corresponding regression equations and R2 values have been explicitly added to the captions of Figures 12 and 13 to ensure quantitative reproducibility. We have also confirmed that all annotations are clearly visible in the revised PDF.

9.Comment: Figure 11 now includes significance letters (a-d) – good. However, the caption should explain that different letters indicate statistically significant differences (p < 0.05) based on Tukey’s HSD.

Response: Thank you for your helpful comment. We have revised the caption of Fig. 11 to explain the meaning of the significance letters. The revised caption states that different lowercase letters above the bars indicate statistically significant differences among groups according to Tukey’s HSD test (p < 0.05). This revision has been made in the revised manuscript, Lines 386–388.

The revised contents: Fig. 11. Effect of polypropylene fibre content on the tensile strength of frozen loess at -5°C and a loading rate of 1.57 mm/min. Different lowercase letters above the bars indicate statistically significant differences among groups according to Tukey’s HSD test (p < 0.05).

The origin contents: Fig. 11 Effect of Polypropylene Fiber Parameters on the Tensile Strength of Frozen Loess at -5°C and 1.57 mm/min.

10.Table 1: The formatting of temperature ranges (e.g., “-2, -3, -5”) is inconsistent; consider using “−2, −3, −5” with proper minus signs.

Response: Thank you for your helpful comment. We have carefully checked the manuscript and revised the formatting of negative temperature values throughout the text, figures, tables, and captions. The hyphen-minus symbol “-” has been replaced with the proper minus sign “-” where negative temperatures are reported, such as “-2, -3, and -5 °C”. This ensures consistent and correct formatting throughout the revised manuscript.

11.Page 39-40: The text for Figure 15 appears garbled (repeated numbers). Please check the final compiled PDF.

Response: Thank you for your careful comment. We apologize for the formatting problem in the previous compiled PDF. We have carefully checked Pages 39–40 and corrected the garbled text associated with Figure 15. The repeated numbers have been removed, and Figure 15, including its caption and annotations, now displays correctly in the final compiled PDF. We have also rechecked the surrounding text and figure layout to ensure that no similar formatting errors remain.

12.Reference 9: “Unknown” as journal – please update if possible, or keep as is but note that it is an old report.

Response: Thank you for your helpful comment. We carefully checked Reference 9 and confirmed that it is not a journal article but an old technical report published by the U.S. Army Cold Regions Research and Engineering Laboratory. Therefore, we have updated the reference by replacing “Unknown” with the report information. In the revised manuscript, Reference 9 is now cited as an old technical report, namely CRREL Report RR350.

The revised contents: Haynes FD, Karalius JA, Kalafut J. Strain rate effect on the strength of frozen silt. Hanover, NH: U.S. Army Cold Regions Research and Engineering Laboratory; 1975. CRREL Report RR 350. Available from: https://ui.adsabs.harvard.edu/abs/1975sesf.rept.....H

The origin contents: Haynes FD, Karalius JA, Kalafut J. Strain effect on the strength of frozen silt. Unknown. 1975 Dec. Available: https://ui.adsabs.harvard.edu/abs/1975sesf.rept.....H

Attachment Submitted filename: Response_to_reviewers_auresp_2.docx

# Peer review: Decision Letter 2

**DOI:** 10.1371/journal.pone.0350932.r005

**Authors:** Shamshad Alam

20 May 2026

<p>Study on tensile mechanical response and microstructure of polypropylene fiber reinforced loess under freezing

PONE-D-26-00128R2

Dear Dr. He,

We’re pleased to inform you that your manuscript has been judged scientifically suitable for publication and will be formally accepted for publication once it meets all outstanding technical requirements.

Within one week, you’ll receive an e-mail detailing the required amendments. When these have been addressed, you’ll receive a formal acceptance letter and your manuscript will be scheduled for publication.

An invoice will be generated when your article is formally accepted. Please note, if your institution has a publishing partnership with PLOS and your article meets the relevant criteria, all or part of your publication costs will be covered. Please make sure your user information is up-to-date by logging into Editorial Manager at Editorial Manager® and clicking the ‘Update My Information' link at the top of the page. For questions related to billing, please contact billing support.

If your institution or institutions have a press office, please notify them about your upcoming paper to help maximize its impact. If they’ll be preparing press materials, please inform our press team as soon as possible -- no later than 48 hours after receiving the formal acceptance. Your manuscript will remain under strict press embargo until 2 pm Eastern Time on the date of publication. For more information, please contact onepress@plos.org.

Kind regards,

Shamshad Alam, PhD

Academic Editor

PLOS One

Additional Editor Comments (optional):

Reviewers' comments:

Reviewer's Responses to Questions

**Comments to the Author**

1. If the authors have adequately addressed your comments raised in a previous round of review and you feel that this manuscript is now acceptable for publication, you may indicate that here to bypass the “Comments to the Author” section, enter your conflict of interest statement in the “Confidential to Editor” section, and submit your "Accept" recommendation.

Reviewer #2: All comments have been addressed

**********

2. Is the manuscript technically sound, and do the data support the conclusions?

The manuscript must describe a technically sound piece of scientific research with data that supports the conclusions. Experiments must have been conducted rigorously, with appropriate controls, replication, and sample sizes. The conclusions must be drawn appropriately based on the data presented.

Reviewer #2: Yes

**********

3. Has the statistical analysis been performed appropriately and rigorously?

Reviewer #2: Yes

**********

4. Have the authors made all data underlying the findings in their manuscript fully available?

The PLOS Data policy requires authors to make all data underlying the findings described in their manuscript fully available without restriction, with rare exception (please refer to the Data Availability Statement in the manuscript PDF file). The data should be provided as part of the manuscript or its supporting information, or deposited to a public repository. For example, in addition to summary statistics, the data points behind means, medians and variance measures should be available. If there are restrictions on publicly sharing data—e.g. participant privacy or use of data from a third party—those must be specified.

Reviewer #2: Yes

**********

5. Is the manuscript presented in an intelligible fashion and written in standard English?

PLOS ONE does not copyedit accepted manuscripts, so the language in submitted articles must be clear, correct, and unambiguous. Any typographical or grammatical errors should be corrected at revision, so please note any specific errors here.

Reviewer #2: Yes

**********

6. Review Comments to the Author

Please use the space provided to explain your answers to the questions above. You may also include additional comments for the author, including concerns about dual publication, research ethics, or publication ethics. (Please upload your review as an attachment if it exceeds 20,000 characters)

Reviewer #2: The authors have adequately addressed the previous comments and improved the manuscript significantly. The paper is now clear, well organized, and suitable for publication. I recommend acceptance in this revised form.

**********

7. PLOS authors have the option to publish the peer review history of their article (what does this mean?). If published, this will include your full peer review and any attached files.

If you choose “no”, your identity will remain anonymous but your review may still be made public.

**Do you want your identity to be public for this peer review?** For information about this choice, including consent withdrawal, please see our Privacy Policy.

Reviewer #2: **Yes:** Sheeraz Ali

**********

# Editor report: Acceptance letter

**DOI:** 10.1371/journal.pone.0350932.r006

**Authors:** Shamshad Alam

PONE-D-26-00128R2

PLOS One

Dear Dr. He,

I'm pleased to inform you that your manuscript has been deemed suitable for publication in PLOS One. Congratulations! Your manuscript is now being handed over to our production team.

At this stage, our production department will prepare your paper for publication. This includes ensuring the following:

* All references, tables, and figures are properly cited

* All relevant supporting information is included in the manuscript submission,

* There are no issues that prevent the paper from being properly typeset

You will receive further instructions from the production team, including instructions on how to review your proof when it is ready. Please keep in mind that we are working through a large volume of accepted articles, so please give us a few days to review your paper and let you know the next and final steps.

Lastly, if your institution or institutions have a press office, please let them know about your upcoming paper now to help maximize its impact. If they'll be preparing press materials, please inform our press team within the next 48 hours. Your manuscript will remain under strict press embargo until 2 pm Eastern Time on the date of publication. For more information, please contact onepress@plos.org.

You will receive an invoice from PLOS for your publication fee after your manuscript has reached the completed accept phase. If you receive an email requesting payment before acceptance or for any other service, this may be a phishing scheme. Learn how to identify phishing emails and protect your accounts at https://explore.plos.org/phishing.

If we can help with anything else, please email us at customercare@plos.org.

Thank you for submitting your work to PLOS ONE and supporting open access.

Kind regards,

PLOS ONE Editorial Office Staff

on behalf of

Dr. Shamshad Alam

Academic Editor

PLOS One
